# Clinical evaluation of droplet digital PCR in the early identification of suspected sepsis patients in the emergency department: a prospective observational study

**DOI:** 10.3389/fcimb.2024.1358801

**Published:** 2024-06-04

**Authors:** Sen Jiang, Dongyang Zhao, Chunxue Wang, Xiandong Liu, Qian Yang, Xiaowei Bao, Tiancao Dong, Gen Li, Yi Gu, Yangqin Ye, Bingke Sun, Shumin Xu, Xiaohui Zhou, Lieying Fan, Lunxian Tang

**Affiliations:** ^1^ Department of Internal Emergency Medicine, Shanghai East Hospital, Tongji University School of Medicine, Shanghai, China; ^2^ School of Medicine, Tongji University, Shanghai, China; ^3^ Department of Clinical Laboratory, Shanghai East Hospital, Tongji University School of Medicine, Shanghai, China; ^4^ Research Center for Translational Medicine, Shanghai Heart Failure Research Center, Shanghai East Hospital, Tongji University, Shanghai, China

**Keywords:** bloodstream infections, droplet digital PCR, sepsis, emergency department, clinical validation

## Abstract

**Background:**

Rapid and accurate diagnosis of the causative agents is essential for clinical management of bloodstream infections (BSIs) that might induce sepsis/septic shock. A considerable number of suspected sepsis patients initially enter the health-care system through an emergency department (ED), hence it is vital to establish an early strategy to recognize sepsis and initiate prompt care in ED. This study aimed to evaluate the diagnostic performance and clinical value of droplet digital PCR (ddPCR) assay in suspected sepsis patients in the ED.

**Methods:**

This was a prospective single-centered observational study including patients admitted to the ED from 25 October 2022 to 3 June 2023 with suspected BSIs screened by Modified Shapiro Score (MSS) score. The comparison between ddPCR and blood culture (BC) was performed to evaluate the diagnostic performance of ddPCR for BSIs. Meanwhile, correlative analysis between ddPCR and the inflammatory and prognostic-related biomarkers were conducted to explore the relevance. Further, the health economic evaluation of the ddPCR was analyzed.

**Results:**

258 samples from 228 patients, with BC and ddPCR performed simultaneously, were included in this study. We found that ddPCR results were positive in 48.13% (103 of 214) of episodes, with identification of 132 pathogens. In contrast, BC only detected 18 positives, 88.89% of which were identified by ddPCR. When considering culture-proven BSIs, ddPCR shows an overall sensitivity of 88.89% and specificity of 55.61%, the optimal diagnostic power for quantifying BSI through ddPCR is achieved with a copy cutoff of 155.5. We further found that ddPCR exhibited a high accuracy especially in liver abscess patients. Among all the identified virus by ddPCR, EBV has a substantially higher positive rate with a link to immunosuppression. Moreover, the copies of pathogens in ddPCR were positively correlated with various markers of inflammation, coagulation, immunity as well as prognosis. With high sensitivity and specificity, ddPCR facilitates precision antimicrobial stewardship and reduces health care costs.

**Conclusions:**

The multiplexed ddPCR delivers precise and quantitative load data on the causal pathogen, offers the ability to monitor the patient’s condition and may serve as early warning of sepsis in time-urgent clinical situations as ED.

**Importance:**

Early detection and effective administration of antibiotics are essential to improve clinical outcomes for those with life-threatening infection in the emergency department. ddPCR, an emerging tool for rapid and sensitive pathogen identification used as a precise bedside test, has developed to address the current challenges of BSI diagnosis and precise treatment. It characterizes sensitivity, specificity, reproducibility, and absolute quantifications without a standard curve. ddPCR can detect causative pathogens and related resistance genes in patients with suspected BSIs within a span of three hours. In addition, it can identify polymicrobial BSIs and dynamically monitor changes in pathogenic microorganisms in the blood and can be used to evaluate antibiotic efficacy and survival prognosis. Moreover, the copies of pathogens in ddPCR were positively correlated with various markers of inflammation, coagulation, immunity. With high sensitivity and specificity, ddPCR facilitates precision antimicrobial stewardship and reduces health care costs.

## Introduction

Bloodstream infections (BSIs) are the leading cause of infection-related death and are associated with substantial morbidity and mortality (Lamy et al., 2020), particularly those might induce sepsis/septic shock (Timsit et al., 2020). Although sepsis may develop without bacterial invasion into the blood stream, pathogens isolated from blood are often considered to be the major causative agent of the sepsis and are utilized to guide the antibiotic treatment. Early detection and effective administration of antibiotics are essential to improve clinical outcomes in critical medical situations like sepsis and septic shock. Delaying the administration of effective antibiotics increases patient mortality, lowers treatment success rates, and raises total healthcare costs (Chertoff and Ataya, 2017; Liu et al., 2017). Therefore, the International Guidelines for Management of Sepsis and Septic Shock 2021 advise using antibiotics right away for adult patients who may have septic shock or sepsis, ideally within one hour of diagnosis to get the optimum therapeutic benefit (Evans et al., 2021). A considerable number of suspected septic patients initially enter the health-care system through an emergency department (ED), hence it is vital to establish an early effective strategy to diagnose sepsis and initiate prompt care for those with life-threatening infection in ED. However, the early identification of the pathogen, timely administration of antibiotics and further improvement of clinical outcomes are often challenging in the ED, due to the lack of development of accurate BSI diagnostic tools for rapid and accurate detection of pathogens.

At present, several BSI prediction tools have been proposed to identify patients at high risk of BSI in ED which include single biomarkers such as C-reactive protein (CRP) (Jeong et al., 2012), serum lactate (Lin et al., 2017) and procalcitonin (PCT) (Hoeboer et al., 2015) or a combination of clinical parameters and biomarkers (Lee et al., 2012; Takeshima et al., 2016; Ljungström et al., 2017). Among all the BSI prediction tools, scores based on clinical parameters and/or bedside biomarkers are proved to be the most rapid way of classifying the risk of BSI and/or sepsis which can be used to guide the diagnostic activities. As a well-known prediction model developed to rule out patients with low risk of positive blood culture (BC), the Shapiro score (SS) (Shapiro et al., 2008) has been widely used, verified, and referenced in the ED worldwide (Hodgson et al., 2016; Jessen et al., 2016; Sparks et al., 2022). More recently, a newly investigation had reported that a Modified Shapiro Score (MSS) was able to predict positive BC in the ED in a well-characterized cohort of patients with suspected BSI and in a subset of patients meeting Sepsis-3 criteria (Nestor et al., 2021).

Apart from early screening out the potential candidates for the suspected patients at high risk of BSI, the precise identification of pathogens also remains a big challenge in the ED and the quantity of pathogen in the blood is crucial for the diagnosis of BSIs. Currently, BC combined with antibiotic susceptibility testing (AST) remains the gold standard for BSI diagnosis, while on the other hand, which is hampered by its low sensitivity and high turnaround time. Emerging molecular diagnostic approaches are used to compensate for the deficiencies of BC, which may be divided into pathogenic bacteria detection based on positive BC samples and pathogenic bacteria detection in the whole blood. The former comprises multiplex PCR, fluorescence *in situ* hybridization, matrix-assisted laser desorption/ionization time-of-flight mass spectrometry, DNA microarray technology, etc. The latter consists of technologies like real-time quantitative PCR (qRT-PCR), next-generation sequencing (NGS), T2 magnetic resonance detection (Timbrook et al., 2017; Sinha et al., 2018). Unfortunately, several above-mentioned molecular tests have a medium sensitivity/specificity, lack of ASTs and therefore have limited diagnostic value in the clinical environment (Marco, 2017; Zhang et al., 2018; Zboromyrska et al., 2019). Droplet digital PCR (ddPCR) is a third generation of qPCR that employs emulsified micro-droplets suspended in oil to generate thousands of droplets that can be counted and used to quantify nucleic acid targets (Wouters et al., 2020). Recently, ddPCR has shown promising potential in resolving polymicrobial infection because it simultaneously achieves unprecedented high sensitivity (able to detect pathogens at low concentrations as low as 10 CFU/ml), high specificity, and absolute quantification without the need for a standard curve (Abram et al., 2020; Wouters et al., 2020). It has been utilized in several medical applications, including liquid biopsy for cancer monitoring, rejection monitoring following organ transplantation and study of prenatal genetic disorders (Caswell et al., 2020; Sorbini et al., 2021; Boldrin et al., 2022). Till now, a few reports had documented that ddPCR had been utilized to detect bacterial infections in septic patients or patients with a highly suspected BSI in the intensive care units (ICU) (Hu et al., 2021; Zheng et al., 2021; Shao et al., 2022; Wu et al., 2022; Lin et al., 2023), which had shown advantages in identifying polymicrobial BSIs and ability to dynamically monitor changes in pathogenic microorganisms in the blood, therefore might be used to evaluate antibiotic efficacy and survival prognosis. As we all know, the intensive care populations mainly with septic shock are to some extent different from a general ED population of patients with varying severity of disease and with relatively lower mortality rates. Therefore, the ratio and species of pathogen identified in the ED might be distinguished from that in the ICU settings. To date, the ddPCR performance specifically for rapid BSI detection in the setting of ED has not been systematically explored yet. Besides, the relationship of the pathogen load detected by ddPCR with the inflammatory and prognostic markers had not been documented and the cost effective of ddPCR had not been studied before. Hence, in the present study, in a prospective cohort of patients with suspected BSIs screened by MSS score in the ED, we comprehensively evaluated the clinical diagnostic application of ddPCR-based methods and made a comparison against the traditional BC as the gold standard. Further, we explored the clinical application value of ddPCR in patients with suspected sepsis.

## Materials and methods

### Study design and patients

This is a single-center prospective observational study, which was conducted in the Department of Internal Emergency Medicine, Shanghai East Hospital, Tong Ji University from 25 October 2022 to 3 June 2023. Based on the MSS scoring system, patients with suspected BSIs in the ED were included. The inclusion criteria were (1) age ≥ 18 years, regardless of sex, (2) MSS≥2 score, (3) written informed consent obtained. The exclusion criteria were (1) age < 18 years, regardless of sex, (2) malignant tumor, HIV patients or any terminal-stage disease, (3) known pregnancy or lactation, (4) participation in other clinical trials, (5) inadequate clinical information or missing experimental data, (6) no signed informed consent obtained. If the inclusion criteria were met, two sets of blood cultures (both aerobic and anaerobic bottles, 10–15ml per bottle) and at least 2ml whole blood samples (EDTA blood collection tubes) were obtained synchronously from the same catheter or vein puncture for BSI diagnosis and ddPCR assay. Other examination including blood routine, blood biochemistry tests, coagulation index, autoimmune antibody, and conventional pathogenic means such as blood smear, serologic tests, and nucleic acid amplification assay were conducted according to our clinical demand. The study allowed the inclusion of multiple episodes of suspected BSI occurring in one patient. This clinical study was conducted after receiving approval from the Ethics Committee of Shanghai East Hospital and was registered on the Chinese Clinical Trial Registry (No. ChiCTR2200065015). All patients or their legal representatives gave written informed consent according to the ethics rules.

### Blood culture, pathogen identification, and antibiotic susceptibility test

The BCs were incubated at 35°C in a BacT/ALERT ^®^ 3D System (bioMérieux, France). When the BC bottle showed a positive signal, smear microscopy was done first, and then the corresponding medium was chosen based on the results of smear Gram staining. When bacteria were detected, the culture was transferred to blood, chocolate, and McConkey agar plates; when fungal hyphae were discovered, the Sabouraud plate was transferred directly. If the anaerobic bacteria were found to be positive, the culture was moved to anaerobic blood agar plate. After overnight incubation, the cultured isolates were identified using matric-assisted laser desorption ionization-time of flight-mass spectrometry (MALDI-TOF MS; Bruker Daltonik GmbH, Bremen, Germany). Then antibiotic susceptibility tests (ASTs) were carried out by a commercial automated VITEK2 COMPACT system (BioMérieux, France) and the results were interpreted according to the Clinical and Laboratory Standards Institute guide-lines(M100-ED30).

### Plasma DNA extraction and ddPCR assay

Each patient’s peripheral venous blood (5 ml) was collected in a tube containing ethylenediaminetetraacetate (EDTA) and promptly centrifuged at 1,200 × g for 5 min. Further, DNA was extracted from 2 ml of plasma using a Magnetic Plasma DNA Kit according to the manufacturer’s protocol (Pilot Gene Technology, Hangzhou, China) (Hu et al., 2021). The DNA was eluted in 50 µl of elution buffer for the following usage. About 40 minutes were needed for sample preparation. On the basis of the latest data of China Antimicrobial Surveillance Network (CHINET)) (Hu et al., 2019) and the common pathogens isolated from our hospital, the designed ddPCR panel consisted of five panels that could identify seven of the most common bacterial pathogens (Pseudomonas aeruginosa, Klebsiella pneumoniae, Escherichia coli, Acinetobacter baumannii, Staphylococcus aureus, Enterococcus, Streptococcus), six fungus (Candida, Pneumocystis jirovecii, Aspergillus, Cryptococcus, Mucor & Rhizopus, talaromyces marneffei), as well as seven antimicrobial resistance genes (blaKPC, mecA, blaOXA-48, blaNDM, blaIMP, vanA, vanM) and five herpes family viruses (herpes simplex virus-1, herpes simplex virus-2, varicella-zoster virus, cytomegalovirus, and Epstein–Barr virus)(**Supplementary Table S1**). Kit instructions and user manuals are available at www.pilotgene.com. Next, a 10 uL ddPCR premix was mixed with 5 uL of plasma DNA. Within 20 minutes, the reaction mixture formed tens of thousands of water-in-oil emulsion droplets via the pressure of the microchannel (Droplet Generator DG32). Finally, the chips were then placed in the thermal cycler TC1 (Pilot Gene Technologies) for 60 minutes of PCR amplification. The cycle settings were 95°C for 5 minutes, 95°C for 15 seconds, and 60°C for 30 seconds, for a total of 40 cycles. The chip scanner CS5 and GenePMS software (v2.0.01.20011) were then used to scan and evaluate droplet count and amplitude data within 30 minutes. The manufacturer’s instructions noted that the target detection threshold for candida, streptococcus, and aspergillus was 1.0 copies/ul, and the threshold for other pathogens was 0.5 copies/ul, with a ddPCR positive defined as higher than the threshold.

### Data collection and clinical adjudication

All data for this study was collected from the electronic medical record system of Shanghai East Hospital using a specific case report for data collection. Data on demographic and clinical characteristics including general clinical profile, blood laboratory examination, isolated pathogens, ddPCR-reported pathogens and DNA load, use of antibiotics, comorbidities, suspected infection site, use of vasoactive drugs, mechanical ventilation, hemodialysis, immunosuppression, hospitalization expenses, antibiotic costs, MSS, Acute Physiological and Chronic Health Assessment II (APACHE II) score, Modified Early Warning Score (MEWS) score, and Sequential Organ Failure Assessment (SOFA) score were collected. Moreover, 28-day mortality rate was recorded. Suspicious infection cases from traditional microbial reports and other detection techniques, such as BC, nasopharyngeal swabs, sputum culture, midstream urine culture, alveolar lavage fluid, tissue/liquid culture, were collected within 7 days of enrollment. Two ED doctors independently verified the outcomes of the ddPCRs and BCs.

**Figure 1:**
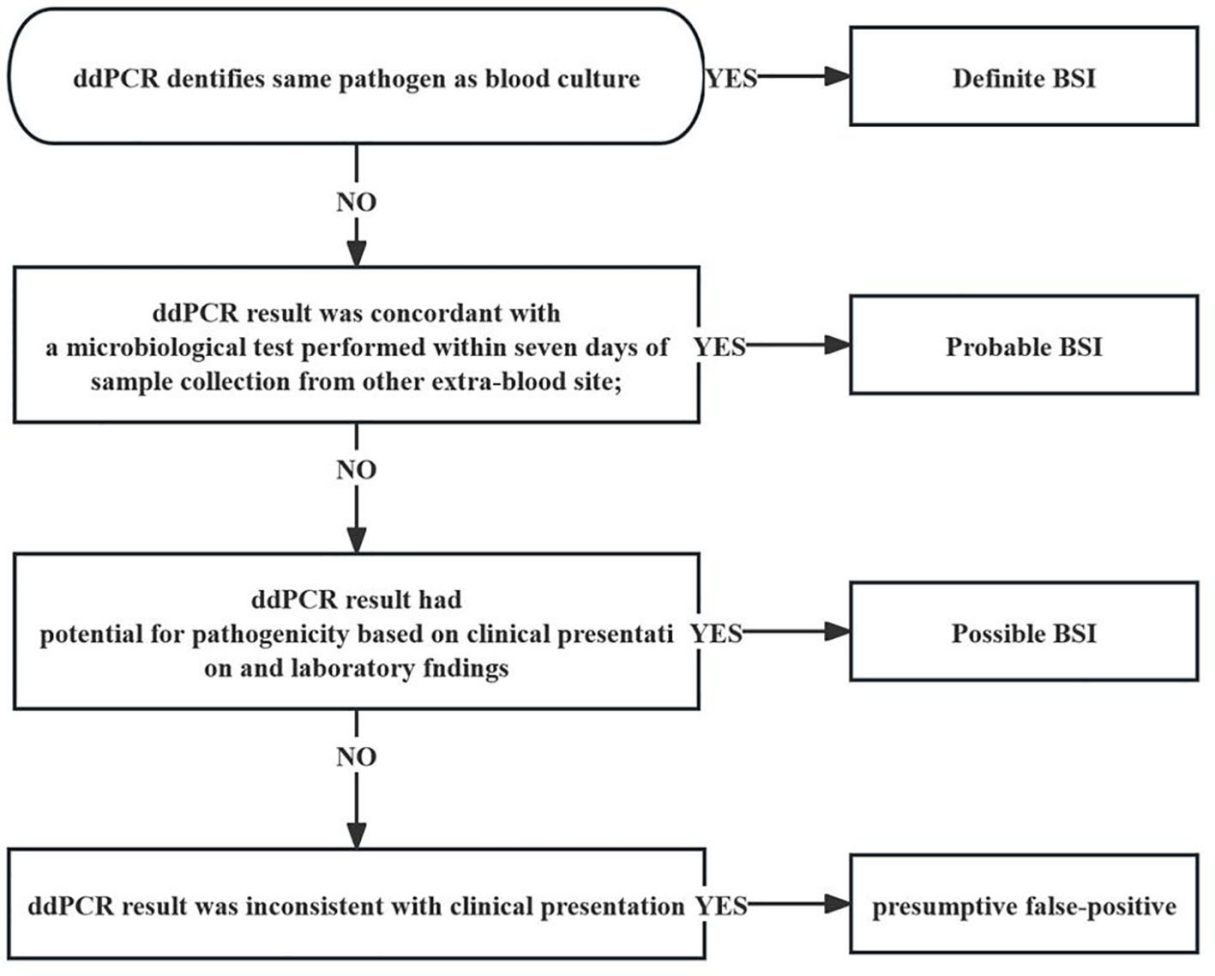
Flow diagram demonstrating definite interpretation of patients with BSI in the cohort. ddPCR, droplet digital PCR; BSI bloodstream infection.

Afterwards, clinical adjudication was conducted separately by the same two adjudicators. According to the standardized algorithm (**Figure 1**), the composite standard of BSI was defined by analyzing all laboratory test results, radiological test results and clinical judgment (Blauwkamp et al., 2019; Kalligeros et al., 2020). The interpretation of ddPCR test results should follow the principles including: (1) being combined with clinical findings, laboratory results and imaging manifestations; (2) referring to other traditional microbial reports and being cross-validated with alter microbiology data; (3) on the basis of the species and copies of microorganisms detected to determine pathogenic bacteria, colonizing bacteria or background bacteria (Wu et al., 2022). The following standardized criteria were used to classify inconsistent cases: (1) definite BSI: ddPCR identified same pathogen as BC; (2) probable BSI: ddPCR result was concordant with a microbiological test performed within seven days of sample collection from other extra-blood site; (3) possible BSI: ddPCR result had potential for pathogenicity based on clinical presentation and laboratory findings; (4) presumptive false-positive: ddPCR result was inconsistent with clinical presentation.

### Statistical analysis

SPSS 23.0 (IBM Corp., Armonk, NY, USA) was used for data analysis. All data were first tested for normality and homogeneity of variance. Normally distributed continuous variables were presented as mean ± standard deviation (SD). Independent samples t-test was used for comparison between two groups, and single-factor analysis of variance was used for comparison between multiple groups. Non-normally distributed continuous variables were expressed as median (quartile) [M (QL, QU)] and analyzed using the Mann-Whitney U test. The chi-square was used to analyze categorical variables, which were expressed as frequencies and percentages. A p value less than 0.05 was considered statistically significant.

## Results

### Clinical characteristics of recruited patients

**Figure 2:**
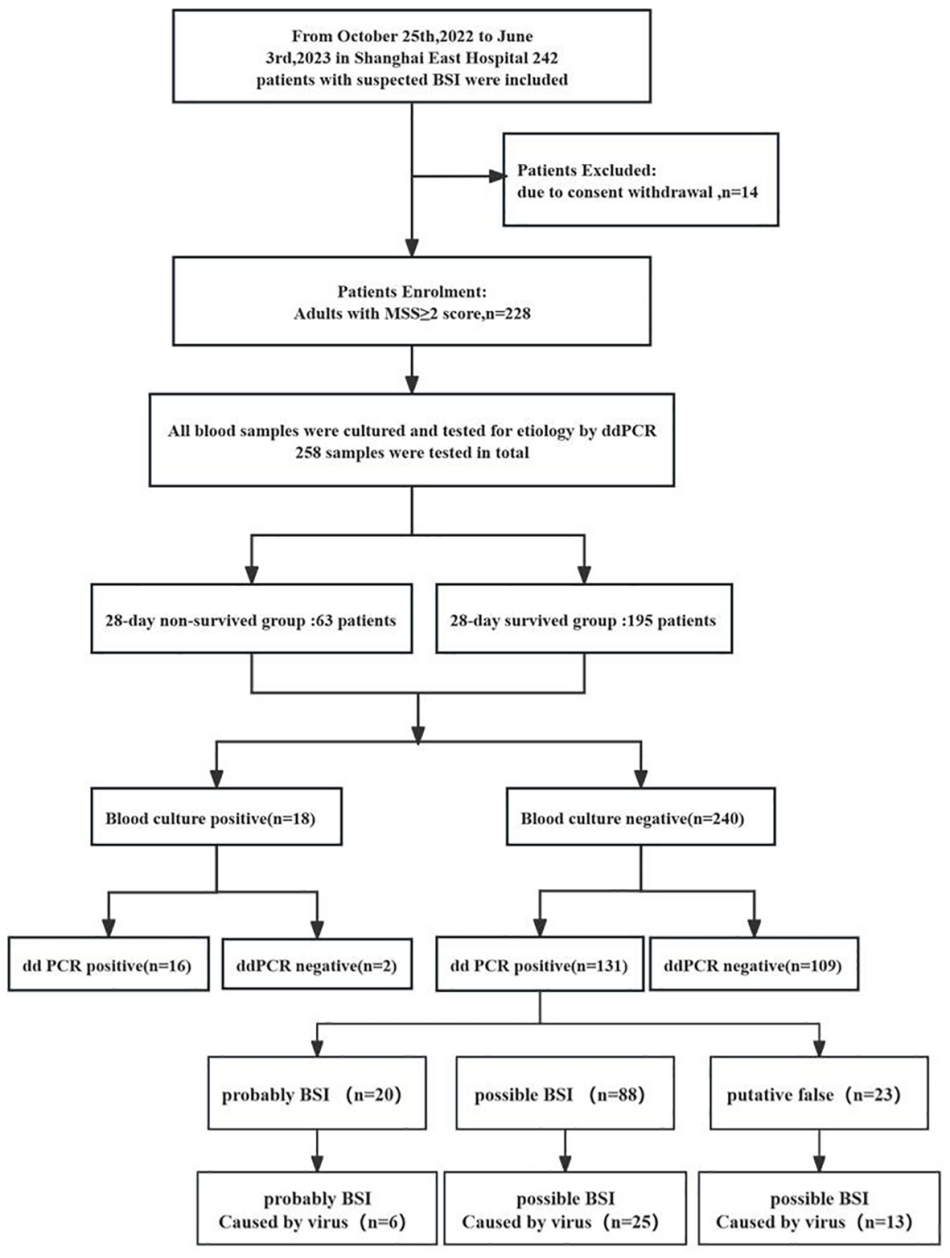
Flow chart for patient enrollment and results analysis. MSS, Modified Shapiro Score, ddPCR droplet digital PCR.

A study outline is shown in **Figure 2**. A total of 258 samples, consisting of BCs and ddPCR performed simultaneously, were collected from 228 patients from October 2022 to June 2023. In these samples, Among them, 205 patients contributed to one samples, 16 patients contributed to two samples, and three sample were collected from other remaining patients. As presented in **Table 1**, the median age of the patients was 78 years (IQR, 70–85 years), and 55.04% (142) were male. In terms of inflammatory indicators, the average plasma levels of CRP, Interleukine-6 (IL-6) and PCT were 61.56mg/L (IQR, 23.17–104.47 mg/L), 34.12 pg/L (IQR, 12.28–103.945 pg/L) and 0.47 ng/L (IQR, 0.106–2.28 ng/L), respectively. In view of coagulation function, the levels of fibrinogen and D-dimer were 4.47g/L (IQR, 3.24–5.75 g/L) and 2.1mg/L (IQR, 1.045–4.83 mg/L), respectively. Moreover, the platelet (PLT) was recorded as 187.05 ± 94.85 10^9/L. The severity of the disease was also assessed on Day 1, with the mean SOFA and APACHE II scores were 3.36 ± 3.27 and 13.01 ± 6.3, respectively. Among these patients with a cumulative 28-day mortality rate of 24.42%, 27.52% experienced acute kidney injury (AKI), 1.6% required renal replacement therapy (RRT), and 17.1% needed mechanical ventilation. Furthermore 15.1% received vasopressors and 66.7% were treated with combination antibiotic therapy. In addition, analysis of the 28-day survivors and non-survivors revealed no significant difference in the prevalence of hypertension and diabetes (P > 0.05). However, the survivors exhibited a younger age (P < 0.01), lower prevalence of AKI and coronary heart disease, and a reduced need of mechanical ventilation (P < 0.001), RRT (P = 0.018), vasoactive drug usage (P < 0.001), and immunosuppression (P < 0.001) when compared with non-survivors (**Table 1**).

**Table 1:** Clinical baseline characteristics of patients with BSIs.

Characteristics	All	28-day survivors	28-day non-survivors	p value
**number**	258	195	63	–
General characteristics				
Age, years	76.12 ± 12.32	74.43 ± 13.02	81.37 ± 7.90	<0.001
Male, n(%)	142 (55.04)	109 (55.9)	33 (52.4)	0.626
Comorbidities				
Hypertension, n (%)	141 (54.65)	108 (55.4)	33 (52.4)	0.677
Diabetes, n (%)	106 (41.09)	83 (42.6)	23 (36.5)	0.396
Coronary heart disease, n (%)	149 (57.75)	104 (53.3)	45 (71.4)	0.011
AKI n (%)	71 (27.52)	39 (20.0)	32 (50.8)	<0.001
Suspected infection site				
Lower respiratory tract, n (%)	210 (81.4)	154 (79.0)	56 (88.9)	0.079
Urinary tract, n (%)	63 (24.42)	49 (25.1)	14 (22.2)	0.641
Intra-abdominal infection, n (%**)**	30 (11.63)	21 (10.8)	9 (14.3)	0.449
Skin and soft tissue, n (%)	15 (5.81)	10 (5.1)	5 (7.9)	0.408
Abscess, n (%)	13 (5.04)	12 (6.2)	1 (1.6)	0.155
Clinical scores				
MSS score, mean (SD)	2.81 ± 1.03	2.53 ± 0.77	3.68 ± 1.25	<0.001
APACHEII score, mean (SD)	13.01± 6.3	11.22 ± 4.86	18.66 ± 6.97	<0.001
SOFA score, mean (SD)	3.36 ± 3.27	2.31 ± 1.96	6.66 ± 4.23	<0.001
MEWS score, mean (SD)	2.09 ± 1.48	1.66 ± 0.98	3.58 ± 1.89	<0.001
Blood laboratory examination				
WBC(10^9/L), mean (SD)	10.11 ± 6.32	9.34 ± 6.07	12.57 ± 6.54	<0.001
Neutrophil percentage(%), mean (SD)	76.86 ± 12.38	74.92 ± 12.06	83.17 ± 11.25	<0.001
Neutrophil cell count(10^9/L)	6.72 (3.9,11.15)	5.98 (3.69,9.21)	9.1 (6.23,14.25)	0.045
Lymphocyte percentage(%)	12.6 (7.2,18.8)	13.9 (7.9,20.1)	8.6 (4.25,14.6)	<0.001
Lymphocyte cell count(10^9/L)	1.005 (0.66,1.435)	1.04 (0.71,1.51)	0.87 (0.465,1.275)	0.178
PLT(10^9/L), mean (SD)	187.05 ± 94.85	195.75 ± 90.63	159.25 ± 103.16	0.008
RDW(%), mean (SD)	14.37 ± 2.79	14.10 ± 2.74	15.26 ± 2.80	0.004
Neutrophil Lymphocyte count ratio (NLCR)	6.02 (3.68,11.73)	5.52 (3.22,10.38)	9.7 (5.5,21.5)	<0.001
PLR	171.57 (113.43,248.69)	172.28 (116.59,256.7)	162.26 (90.96,243.1)	0.678
RPR	0.08 (0.06,0.12)	0.08 (0.06,0.1)	0.11 (0.07,0.19)	<0.001
CRP(mg/L)	61.56 (23.17,104.47)	55.27 (20.44,99.62)	69.19 (34.83,144.97)	0.003
IL-6(pg/mL)	34.12 (12.28,103.95)	26.41 (10.86,76.92)	75.32 (22.02,221.2)	<0.001
SAA(mg/L)	149.119 (56.82,288)	151.67 (52.23,288)	143.05 (62.41,219.12)	0.670
PCT(ng/mL )	0.47 (0.11,2.28)	0.28 (0.09,1.66)	1.47 (0.49,5.97)	0.694
HBP(ng/mL)	59.99 (27.33,150.64)	59.84 (26.72,144.05)	61.64 (29.95,156.68)	0.860
Lac(mmol/L)	2 (1.6,2.6)	1.9 (1.5,2.4)	2.4 (1.8,3.4)	0.616
SCr(umol/L), mean (SD)	101.56 ± 74.91	85.34 ± 43.39	152.39 ± 118.57	<0.001
ESR(mm/h), mean (SD)	41.53 ± 24.35	43.45 ± 24.94	32.20 ± 19.34	0.103
Ferritin(ug/L)	492 (285,767)	477 (184.5,778)	541.5 (305.25,750)	0.935
IL-1β(pg/mL)	2.5 (2.5,2.66)	2.5 (2.5,2.68)	2.5 (2.5,2.59)	0.897
IL-2(pg/mL)	2.5 (2.5,2.5)	2.5 (2.5,2.5)	2.5 (2.5,2.5)	0.370
IL-4(pg/mL)	2.5 (2.5,2.55)	2.5 (2.5,2.5)	2.5 (2.5,2.98)	0.371
IL-5(pg/mL)	2.5 (2.5,2.5)	2.5 (2.5,2.5)	2.5 (2.5,2.5)	0.193
IL-8(pg/mL)	17.17 (8.94,41.68)	14 (7.49,30.5)	37.67 (15.65,87.47)	<0.001
IL-10(pg/mL)	4.77 (2.97,8.82)	4.44 (2.73,7.44)	7.16 (4.18,14.68)	0.253
IL-12P70(pg/mL)	2.5 (2.5,2.68)	2.5 (2.5,2.54)	2.5 (2.5,2.83)	0.360
IL-17(pg/mL)	10 (10,14.74)	10 (10,14.79)	10 (10,14.09)	0.270
IFN-α(pg/mL)	2.5 (2.5,2.5)	2.5 (2.5,2.5)	2.5 (2.5,2.5)	0.909
IFN-γ(pg/mL)	2.57 (2.5,3.95)	2.64 (2.5,3.99)	2.5 (2.5,3.82)	0.414
TNF-α(pg/mL)	2.5 (2.5,3.09)	2.5 (2.5,3.17)	2.5 (2.5,2.81)	0.643
CD3(uL), mean (SD)	619.65 ± 381.86	641.89 ± 377.35	551.88 ± 391.95	0.181
CD4(uL), mean (SD)	398.83 ± 278.92	399.15 ± 261.83	397.86 ± 329.02	0.979
CD8(uL), mean (SD)	193.01 ± 136.07	210.23 ± 143.91	140.56 ± 91.79	0.003
CD4/CD8(%)	1.98 (1.31,3.63)	1.9 (1.3,3.17)	2.2 (1.6,4.4)	0.231
CD19(uL)	106.5 (59.25,204)	122 (61,217)	92 (49,178)	0.347
CD16+CD56+(%)	157.5 (87.75,263)	170 (92,260)	107 (66,274)	0.930
HLA-DR+CD3+/CD3+(%), mean (SD)	40.56 ± 21.05	41.84 ± 21.67	35.41 ± 17.93	0.212
Regulatory T cell (%), mean (SD)	3.16 ± 1.54	3.16 ± 1.58	3.16 ± 1.40	0.993
nCD64 index	5.84 (1.21,28.67)	9.15 (1.14,32.88)	1.77 (1.25,8.55)	0.037
C1q(mg/L ), mean (SD)	157.09 ± 42.58	161.69 ± 41.82	141.10 ± 41.82	0.005
C3(mg/L )	0.98 (0.83,1.25)	1.065 (0.85,1.28)	0.88 (0.67,1.03)	0.484
C4(mg/L ), mean (SD)	0.28 ± 0.13	0.29 ± 0.14	0.27 ± 0.13	0.489
IgG (g/L), mean (SD)	11.45 ± 3.52	11.54 ± 3.49	11.20 ± 3.64	0.624
IgA (g/L), mean (SD)	2.79 ± 1.29	2.69 ± 1.24	3.05 ± 1.39	0.157
IgM (g/L), mean (SD)	0.8 ± 0.45	0.86 ± 0.49	0.64 ± 0.28	0.012
IgE (g/L)	91.49 (23.78,210.38)	94.81 (24.89,220.95)	71.29 (21.22,167.83)	0.774
NGAL(ng/mL)	87 (70.5,98.5)	82 (62,91.5)	842 (99,1114)	0.636
TAT(ng/mL)	4.06 (2.56,6.84)	3.75 (2.31,6.25)	6.24 (3.51,7.88)	0.079
tPAIC (ng/mL)	5.16 (3.09,8.24)	4.88 (2.98,7.55)	10.42 (4.06,25.46)	<0.001
TM(TU/mL), mean (SD)	15.04 ± 7.8	14.16 ± 7.01	20.43 ± 10.17	0.001
PIC (ug/mL)	1.33 (1,1.92)	1.32 (1.00,1.93)	1.33 (0.93,1.78)	0.637
D-dimer (mg/L)	2.1 (1.05,4.83)	1.68 (0.94,4.00)	4.45 (2.17,8.39)	0.004
Fibrinogen (g/L), mean (SD)	4.58 ± 1.73	4.77 ± 1.70	3.97 ± 1.69	0.002
APTT (g/L), mean (SD)	31.41 ± 8.42	30.32 ± 6.99	34.94 ± 11.31	<0.001
PT (S), mean (SD)	14.34 ± 4.86	13.94 ± 4.56	15.62 ± 5.59	0.019
Clinical characteristics during hospitalization				
Renal replacement therapy, n(%), mean (SD)	4 (1.6)	1 (0.5)	3 (4.8)	0.018
Use of vasoactive drugs, n(%), mean (SD)	39 (15.1)	8 (4.1)	31 (49.2)	<0.001
Mechanical ventilation, n(%), mean (SD)	44 (17.1)	19 (9.7)	25 (39.7)	<0.001
Combination antibiotic therapy, n(%), mean (SD)	172 (66.7)	115(59.0)	57 (91.9)	<0.001
Immunosuppression, n(%), mean (SD)	76 (29.5)	38 (19.6)	38 (61.3)	<0.001
Outcomes				
Hospitalization expenses (RMB)	42275.04 (26497.07,86109.64)	38839.34 (25408.35,58302.21)	88737.45 (33113.53,193645)	<0.001
Antibiotic costs (RMB)	5127.63 (2643.1,10311.25)	4678.245 (2575.923,7935.403)	8952.88 (2857.26,45982)	<0.001
hospital stay, n (%)	14 (10,21.5)	14 (10,19)	13.5 (10,28.75)	0.004
ICU days, n (%),	0 (0,4.5)	0 (0,0)	2.5 (0,13)	<0.001

Data were expressed as a mean ± standard deviation for normally distributed continuous variables or median (interquartile range) for non-normally distributed continuous variables. Categorical variables were expressed as n (%).

AKI, acute kidney injury; WBC, white blood cell; PLT, platelet; RDW, red blood cell volume distribution width; CRP, C-reactive protein; IL, Interleukin; SAA, Serumamyloid A; PCT, procalcitonin; HBP, Heparin-Binding Protein; ESR, erythrocyte sedimentation rate; tPAIC, tissue Plasminogen Tctivator-inhibitor Complex; TM, thrombomodulin; PT, prothrombinTime; APTT, activated partial thromboplastin time; APACHE II, Acute Physiology and Chronic Health Evaluation II; SOFA, Sequential Organ Failure Assessment; MSS, Modified Shapiro Score; ddPCR, droplet digital PCR.

### Performance of the ddPCR testing and the concordance between ddPCR and BC

**Figure 3:**
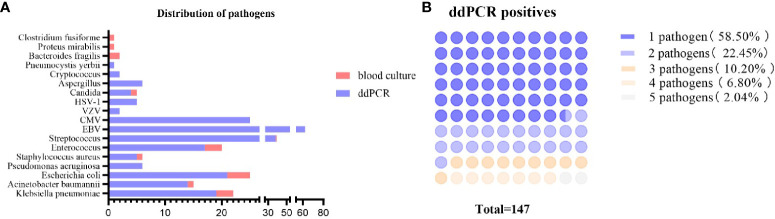
Distribution of pathogens detected by blood culture and ddPCR testing. **(A)** Pathogens detected by ddPCR and blood culture. Blue bars represent the episodes in which the pathogens were detected by ddPCR, orange bars mean that the pathogens were detected by blood culture. The length of the bar represents the number of episodes. **(B)** Counts and percentages of co-infections in patients of ddPCR-positive. ddPCR droplet digital PCR.

In general, as illustrated in **Table 2**; **Figure 3**, the etiological diagnosis revealed that the ddPCR yielded 103 positive results from a total of 214 blood samples, with a positive rate of 48.13%. Among them, bacteria accounted for 90.15% and 9.85% for fungi (**Table 2**). Of all the bacteria detected, the proportion of Gram-positive (G^+^) bacteria and Gram-negative(G^-^) bacteria were 49.58% and 50.42%, respectively. In contrast, BC only detected 18 positives. Among all pathogens detected by ddPCR, Streptococcus (n = 38) were the most frequently identified. Moreover, 60 G^-^ bacteria were detected, with the top three strains being E. coli (n = 21), K. pneumoniae (n = 19), and A. baumannii (n = 14). Furthermore, the ddPCR assay revealed the presence of 59 G^+^ pathogens, with Streptococcus (n = 38), Enterococcus (n = 16), and Staphylococcus aureus (n =5) being the predominant species. Additionally, Candida (n= 5) and Aspergillus (n = 6) were the most frequently detected fungi. As shown in **Table 2**; **Supplementary Table S2**; **Figure 3**, results of BC and ddPCR were concordantly positive in 16 episodes with 13 identical pathogens and 3 different pathogens, concordantly negative in 109 episodes, and discordant in 124 episodes. In comparison with BCs, with the most common causative agents of culture-proven BSI being Escherichia coli (22.2%), Klebsiella pneumoniae (16.7%), Enterococcus (16.7%), and Staphylococcus aureus (5.56%), pathogens included in the ddPCR panel were identified in 88.8% (16 out of 18) of positive BCs (**Supplementary Table S2**). Notably, the target detection listed by ddPCR did not encompass Clostridium fusiforme, Bacteroides fragilis, and Proteus mirabilis, which were identified through BC.

**Table 2:** Performance of ddPCR results for targeted organisms.

	BC+/ddPCR+,n	BC+/ddPCR-,n	BC-/ddPCR+,n	BC-/ddPCR-,n
Pathogens (all)	13	5	119	109
Klebsiella pneumonia	3	0	16	–
Acinetobacter baumannii	1	0	13	–
Escherichia coli	3	1	18	–
Pseudomonas aeruginosa	0	0	6	–
Staphylococcus aureus	1	0	4	–
Enterococcus	3	0	13	–
Streptococcus	1	0	37	–
Candida	1	0	4	–
Aspergillus	0	0	6	–
Pneumocystis jiroveci	0	0	1	–
Cryptococcus	0	0	1	–
Clostridium fusiforme	0	1	0	–
Bacteroides fragilis	0	2	0	–
Proteus mirabilis	0	1	0	**-**

EBV, epstein-barr virus; CMV, cytomegalovirus; VZV, varicella-zoster virus; HSV-1, Herpes simplex virus 1.

**Figure 4:**
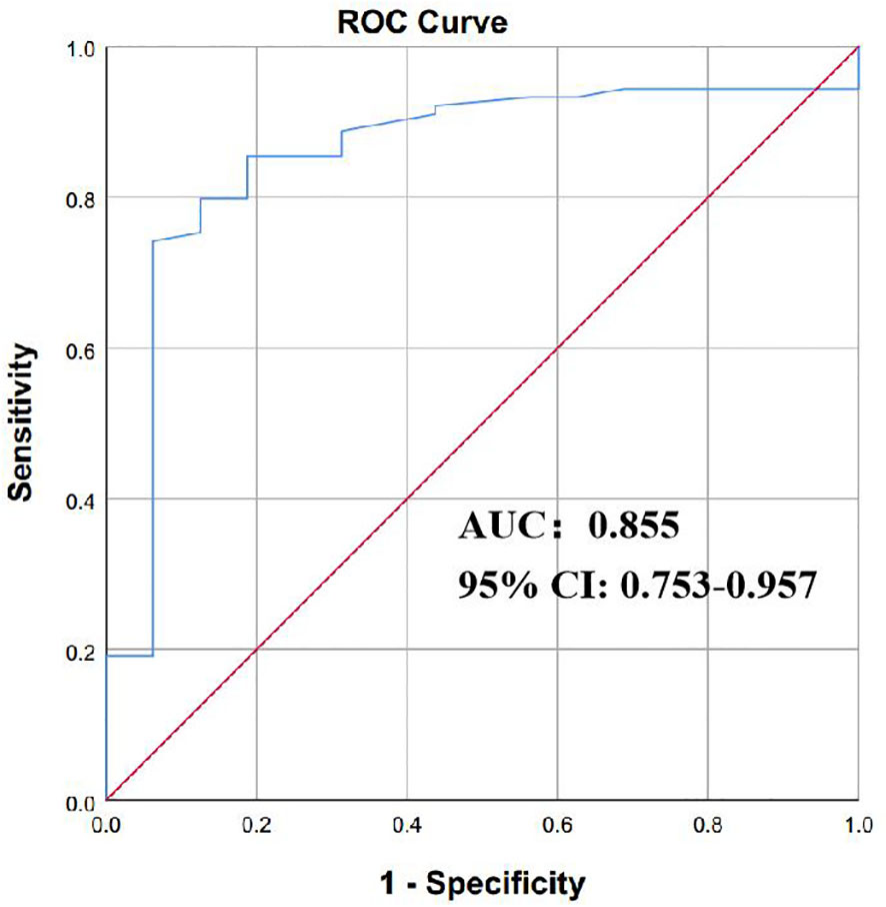
The efficacy of ddPCR in diagnosing of BSI. The receiver operating characteristic curves (ROC) of ddPCR for diagnosis of bloodstream infection. P values were calculated using log-rank tests. ddPCR droplet digital PCR; BSI bloodstream infection.

On the basis of BC testing, the calculation principle was the aggregate ddPCR detection, demonstrating a sensitivity of 88.89%, a specificity of 55.61%, a positive predictive value (PPV) of 15.53%, and a negative predictive value (NPV) of 98.2%; for clinical diagnosis-proven BSIs, the sensitivity and specificity are 84.54% and 55.61%, respectively (**Table 3**). The optimal diagnostic power for quantifying BSI through ddPCR is achieved with a copy cutoff of 155.5, which strikes a balance between sensitivity in detecting positive BSI patients and specificity in identifying case controls. The area under the receiver operating characteristic (AUROC) curves was determined to be 0.855 [95% confidence interval 0.753–0.957] (**Supplementary Table S3**; **Figure 4**). These preliminary data suggested that ddPCR had potential to rapidly identify targeted pathogens with high specificity and specificity.

**Table 3:** Sensitivity and Specificity of ddPCR in detecting different types of pathogens.

sample(n=214)	ddPCR+	ddPCR-	Sensitivity(%)	Specificity(%)	PPV(%)	NPV(%)
Positive by blood culture	16	2	88.89	55.61	15.53	98.20
Negative by blood culture	87	109				
Positive by all microbiological testing	30	10	75.00	58.05	29.13	90.99
Negative by all microbiological testing	73	101				
Positive by clinical diagnosis	93	17	84.54	90.38	90.29	84.68
Negative by clinical diagnosis	10	94				

ddPCR, droplet digital PCR; PPV, positive predictive value; NPV, negative predictive value; G-, gram-negative bacteria; G+, gram-positive bacteria.

In addition to pathogen identification, the AMR genes panel was utilized to identify seven AMR genes, namely blaKPC, mecA, blaOXA-48, blaNDM, blaIMP, vanA, and vanM. However, only the blaKPC, mecA, and blaNDM genes were found to be positive through ddPCR testing, as shown in **Table 4**. The ddPCR analysis revealed that there were 5 episodes with a positive result for blaKPC and 1 for blaNDM. Among these episodes, the simultaneous detection of K. pneumoniae and the AMR gene occurred in 31.6% of cases, which held significant clinical implications. In comparison to the results obtained from the BC, it was observed that two instances of K. pneumoniae reported in the BC exhibited resistance towards carbapenems. Furthermore, the ddPCR results indicated the presence of the bla KPC gene in these strains. Notably, the mecA positive sample and blaNDM positive sample were not subjected to pathogen testing. The appearance of the plasmid gene blaNDM might be due to the different stability between bacterial cfDNA and cell-free plasmids. In contrast to linear genomic DNA, secondary structures may play an important role in protecting plasmid DNA from nuclease degradation (Gill et al., 2009). However, gene mecA is usually located in the Staphylococcal chromosome. It is likely that low circulating DNA concentrations in false-negative samples were below the limit of detection of the assay (Khier and Lohan, 2018). From a therapeutic respective, the identification of drug resistance genes within a span of three hours facilitated the selection of sensitive antibiotics for the target pathogen as determined by the initial day ddPCR assay. Consequently, the patient’s condition exhibited gradual improvement, accompanied by a reduction in both pathogen load and AMR gene load (**Supplementary Table S4**).

**Table 4:** AMR genes detected by ddPCR and the related pathogens.

AMR genes	Pathogens	counts
blaKPC(n=5)	Klebsiella pneumoniae	5
blaNDM(n=2)	Klebsiella pneumoniae	1
	None	1
mecA (n=10)	Staphylococcus aureus	3
	None	7

### Clinical potential value of ddPCR for Epstein-Barr virus infection

**Figure 5:**
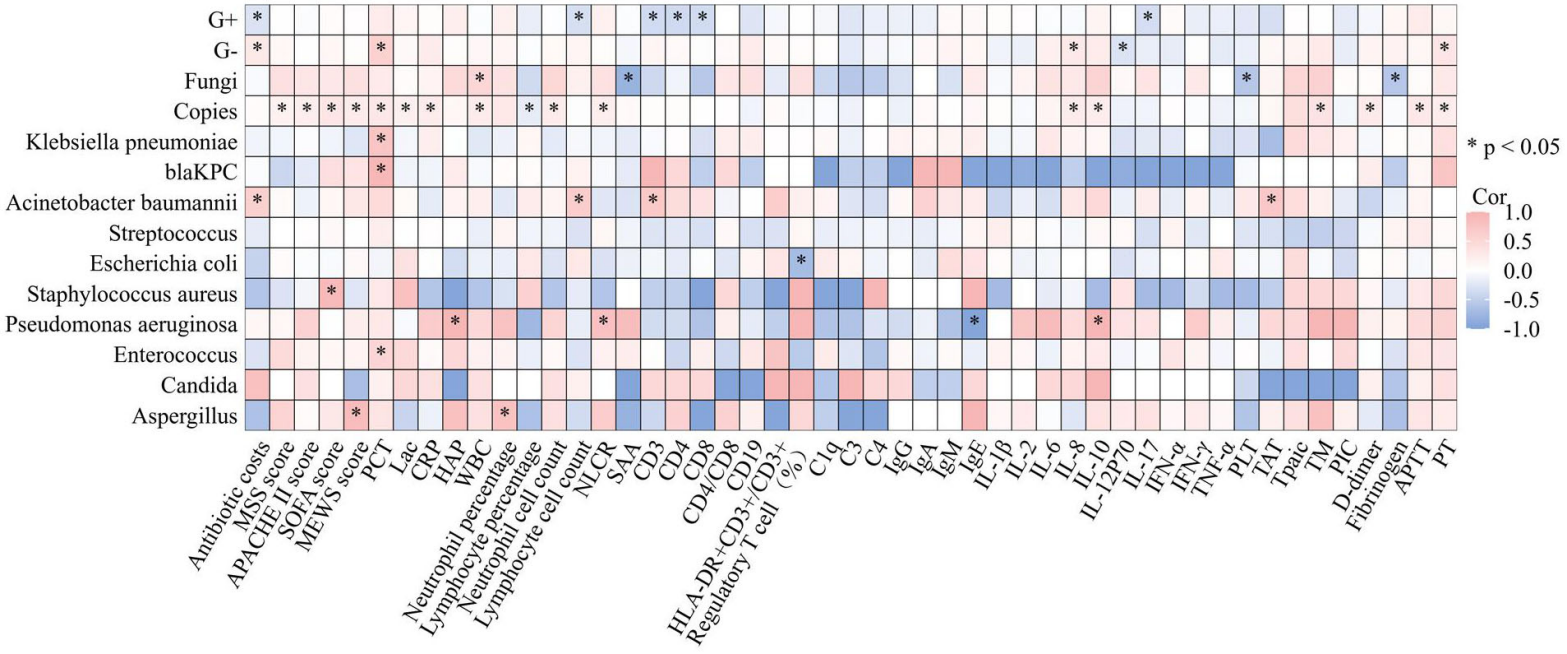
Correlations between copies of pathogens detected by ddPCR and clinical characteristics. Heatmap shows the correlation between the copies of pathogens identified through ddPCR in BSIs and various markers of inflammation, coagulation, immunity and prognosis. *p<0.05, APACHE, II Acute Physiology and Chronic Health Evaluation II; SOFA, Sequential Organ Failure Assessment; MSS, Modified Shapiro Score; PLT platelet; RDW red blood cell volume distribution width; CRP, C-reactive protein; IL, Interleukin; SAA, Serumamyloid A; PCT, procalcitonin; HBP Heparin-Binding Protein; ESR, erythrocyte sedimentation rate; tPAIC, tissue Plasminogen Tctivator-inhibitor Complex; TM, thrombomodulin; PT, prothrombinTime; APTT, activated partial thromboplastin time; ddPCR, droplet digital PCR; G+, bacteria Gram-positive bacteria; G−, bacteria Gram-negative bacteria.

EBV was the most frequently identified virus in our study. As shown in **Table 5**, a total of 258 episodes from 228 patients with BSIs underwent testing for EBV antibody and mcfDNA using ddPCR. Of these, 69(26.74%) tested positive for EBV reactivation. Among these 69 cases, 35(50.72%) were found to have concurrent COVID-19 infection and 21(30.43%) were accompanied with immunosuppression. In addition, the 28 day survival rate is 34.78% (**Supplementary Table S5**). When it comes to EBV antibody, we found that the EBV antibodies (VCA-IgM, VCA-IgG, and EBNA-IgG) in blood were related to copy number of ddPCR in BSI patients. Our results indicated that the copy number of ddPCR with VCA-IgM negative, VCA-IgG negative, and EBNA-IgG negative in blood was significantly higher than that of VCA-IgM negative, VCA-IgG positive, and EBNA-IgG positive. Consequently, the group characterized by VCA-IgM negative, VCA-IgG negative, and EBNA-IgG negative was considered to exhibit immunologic unresponsiveness associated with immunosuppression (**Table 5**). The correlation analysis conducted between the number of EBV copies as determined by ddPCR and immune indicators revealed a statistically significant correlation between the EBV copy number and the CD4+/CD8+ ratio (r = -0.312, p = 0.029) (**Figure 5**).

**Table 5:** EBV detected by ddPCR assay and related comorbidities.

Anti-EBV Antibodies			number	Explanation	EBV(copy number)	COVID-19	Immunosuppression	28 day survival
VCA-IgM	VCA-IgG	EBNA-IgG						
negative	negative	Negative	32	No immune response	879.53 ± 1407.78	12(37.5%)	13(40.63%)	16(50%)
positive	negative	Negative	9	Acute infection	19414.44 ± 24911.87	8(88.89%)	4(44.44%)	3(33.33%)
negative	positive	Positive	26	Previous infection	696.38 ± 866.30	14(53.85%)	3(11.54%)	4(15.38%)
negative	positive	Negative	2	Acute infection/Previous infection	19515 ± 27431.50	1(50%)	1(50%)	1(50%)

VCA, viral capsid antigen; EBNA, Epstein Barr Virus Nuclear Antigen; COVID-19, Corona Virus Disease 2019.

### Clinical potential value of ddPCR for liver abscess

In our research, it was observed that ddPCR exhibited a high level of sensitivity and specificity in detecting liver abscess in patients (**Supplementary Table S6**). The ratio of pathogens detected by ddPCR to those detected by pus culture was found to be 100% (7 out of 7 cases). Among these cases, Klebsiella pneumoniae was detected in 5 episodes, while Escherichia coli was detected in two. In stark contrast BC did not yield any relevant pathogenic bacteria. Moreover, liver abscess patients underwent antimicrobial de-escalation therapy following negative results obtained from the ddPCR assay conducted on the third day. **Supplementary Figure S1** displays scatter plots of liver abscess representative chip analysis results from a clinical case that was dynamically examined and clinically improved after antibiotic treatment.

### Correlative analysis between ddPCR and the biomarkers

Sepsis develops as a consequence of a complicated, dysregulated host response to infection, which is characterized not only by increased inflammation but also mainly by abnormal coagulation function as well as immune suppression. To explore whether the correlation exists between the copies of pathogens identified through ddPCR in BSIs and various markers of inflammation, coagulation, and immunity, spearman correlation coefficient was utilized to describe the relationship. The results are presented in **Figure 5**. From the perspective of inflammatory markers, we observed the correlation between the levels of following inflammatory indicators and the pathogen load identified by ddPCR (PCT: Spearman’s rho = 0.309, P<0.001; CRP: Spearman’s rho = 0.242, P = 0.004; Neutrophil Lymphocyte count ratio (NLCR): Spearman’s rho = 0.221, P = 0.009; White blood cell (WBC): Spearman’s rho = 0.254, P = 0.002; Neutrophil percentage: Spearman’s rho = 0.294 P<0.001; Neutrophil cell count:Spearman’s rho = 0.242 P = 0.004; Lymphocyte percentage: Spearman’s rho = -0.196 P =0.021), while no relationship was found with lymphocyte count. Additionally, a correlation was discovered between the PCT level and the pathogen load of G^-^ bacteria detected by ddPCR (Spearman’s rho = 0.589, P < 0.001). Furthermore, Klebsiella pneumoniae exhibited a correlation with PCT (Spearman’s rho = 0.757, P < 0.001) as well as blaKPC (Spearman’s rho = 0.928, P < 0.01). However, no correlation was found between the pathogen load with heparin-binding protein (HBP) or Serumamyloid A (SAA). Cytokines are important indicators of inflammation, indeed, when considering copies of pathogens, a positive correlation was found with cytokines such as IL-6, IL-8 and IL-10, with correlation coefficients of 0.192, 0.241 and 0.240, respectively. For other cytokines like Interleukin-1β (IL-1β), IL-17, Tumor Necrosis Factor-a (TNF-α), IL-4 and IL-5 were not found to be related to the pathogen load in our research. From the view point of coagulation, the ddPCR assay revealed a significant positive correlation between the copies of pathogens detected and coagulation parameters, including tissue Plasminogen Activator-inhibitor Complex (tPAIC) (Spearman’s rho = 0.421, P < 0.001), thrombomodulin (TM) (Spearman’s rho = 0.364, P< 0.01), D-dimer (Spearman’s rho = 0.271, P< 0.001), ProthrombinTime (PT) (Spearman’s rho = 0.248, P < 0.01), and Activated Partial Thromboplastin Time (APTT) (Spearman’s rho = 0.291, P< 0.01). In the respect of the correlation between the copies of pathogens detected and immunity indicators, it was observed that copies of pathogens were not related to cellular immunity-related markers as CD3, CD4, CD8, Treg, Human Leukocyte Antigen-DR (HLA-DR)/CD3 and humoral immunity-associated proteins as CD19 and IgG, IgA and IgM. We also found no correlations between copies of pathogens and complement system biomarkers as C1q, C3 and C4. As a widely used biomarker reflecting the severity of sepsis, lactate was found to be related to the pathogen load detected by ddPCR (Spearman’s rho = 0.19, P < 0.05).

### ddPCR performance for 28-day prognosis

**Figure 6:**
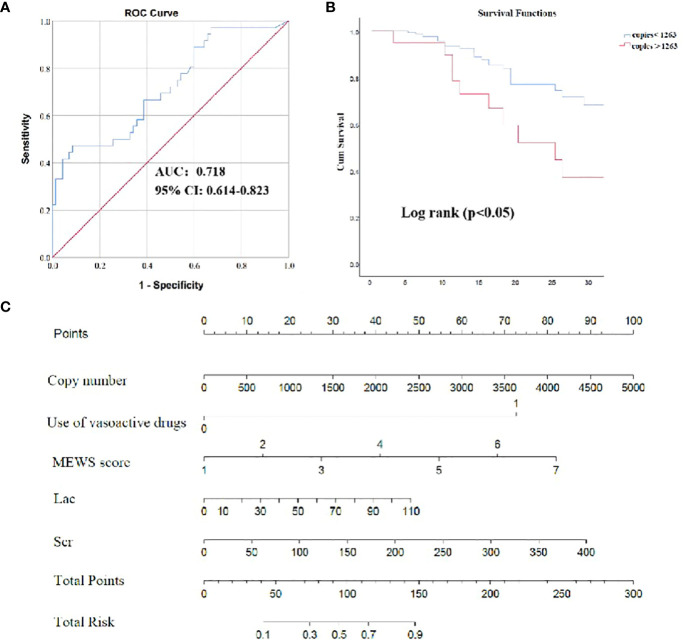
The efficacy of ddPCR in predicting 28-day survival prognosis. **(A)** The receiver operating characteristic (ROC) curve of ddPCR for 28-day survival of BSI. **(B)** Survival curves of patients with BSI according to copies of ddPCR. **(C)** Nomogram to predict the risk of 28-day mortality. P values were calculated using log-rank tests. NLCR, Neutrophil Lymphocyte count ratio.

The aforementioned results revealed that pathogens identified through ddPCR was closely related to indicators reflecting the severity of infection (**Figure 5**). Subsequently, correlation analysis between severity of illness score and pathogen load detected by ddPCR was conducted, and they exhibited a positive correlation (SOFA: Spearman’s rho = 0.322, P< 0.001; APACHE II: Spearman’s rho = 0.217, P< 0.01; MEWS: Spearman’s rho = 0.244, P< 0.01) (**Figure 5**). To assess the performance of ddPCR as a continuous metric in comparison to other established prognostic biomarkers, we conducted calculations of AUROCs for 28-day mortality. As presented in **Supplementary Table S7**; **Figure 6**, our findings indicated that ddPCR exhibited an AUROC of 0.718(95% CI, 0.614–0.823) for the identification and prediction of 28-day prognosis. The result demonstrated a sensitivity of 47.2% and a specificity of 91.4%, with a derived cut-off of 1263 copies/ml. Further analysis revealed that the 28-day mortality rate was 2.215 times higher for those with copies greater than 1263 than those less than 1263 (HR: 2.215, 95%CI: 1.113–4.405). The corresponding Kaplan-Meier curves and the outcomes of log-rank tests for ddPCR copy numbers above or below 1263 copies/ml were also presented. The results of univariate analysis for patients with BSIs who survived for 28 days (n = 195) and those who did not survive for 28 days (n = 63) in the development cohort are presented in **Supplementary Table S8**. Multivariate analysis revealed that lactic acid (Lac) and serum creatinine (Scr) were identified as independent risk factors for the 28-day mortality in patients with BSI in the development cohort (**Table 6**). A nomogram was constructed based on the aforementioned equation. The calibration plot of the nomogram demonstrated a satisfactory fit within the development cohort, when considering the predicted probability or the actual probability. Additionally, it exhibited strong statistical consistency in predicting the 28-day mortality caused by BSI, as evidenced by a C value of 0.805. In comparison with the outcomes observed, the nomogram displayed a sensitivity of 74.4% and a specificity of 76.2% in predicting 28-day mortality (**Supplementary Figure S2**). As we had found that the ddPCR was related to the severity of disease, we further assessed the ICU time and hospital stays between positive and negative groups. To our surprise, we found no statistical difference between two groups (**Supplementary Figure S3**).

**Table 6:** Logistic regression analyses of factors associated with 28-day prognosis focus on ddPCR.

	p	HR	95.0% CI	
			Lower	Upper
copy number	0.037	1.000	1.000	1.000
Use of vasoactive drugs	0.002	4.205	1.681	10.516
MEWS score	0.005	1.312	1.084	1.588
Lac(mmol/L)	0.012	1.026	1.006	1.047
SCr(umol/L)	0.001	1.036	1.014	1.058

MEWS, Modified Early Warning Score; Lac, lactic acid; Scr, Serum creatinine.

### Health economic evaluation of the ddPCR

**Figure 7:**
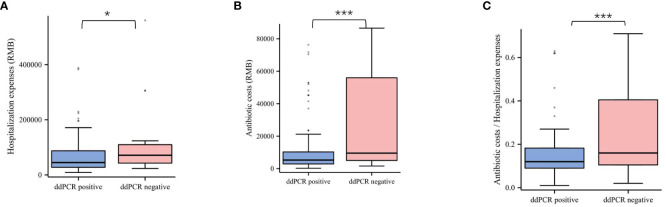
Comparison of the health economic values between negative and positive groups divided by ddPCR assay. **(A)** Comparison of the hospitalization expenses between ddPCR positive and ddPCR negative patients. **(B)** Comparison of the antibiotic costs between ddPCR positive and ddPCR negative patients. **(C)** Comparison of the percentage of antibiotics costs in the total hospitalization expenses between ddPCR positive and ddPCR negative patients. *p<0.05, ***p<0.001, ddPCR, droplet digital PCR.

Considering economic factors of ddPCR for identifying causative pathogens in BSIs, the cost is approximately $150 for ddPCR assays covering common isolated pathogens and AMR genes, and $60 for blood culture in China. Compared with BC, the cost of ddPCR assay are still relatively expensive, while is considerably lower than that of the mNGS. However, our study has shown that the ddPCR assay had several advantages in health economic evaluation which is exhibited in **Figure 7**. Based on the results of the microbiological test and clinical assessment, patients diagnosed with BSI were categorized into negative and positive groups using ddPCR assay. Notably, the negative groups identified through ddPCR exhibited comparatively lower hospitalization expenses when compared to the positive groups identified through ddPCR (P < 0.05). When it comes to the antibiotics cost, negative groups were significantly higher than that of positive groups (P < 0.001). In addition, we evaluated the percentage of antibiotics costs within the total hospitalization expenses, with the result that negative groups was comparatively lower than the positive groups (P<0.001). The rapid and accurate identification of causative pathogens is associated with improved mortality and reduced healthcare costs. Therefore, ddPCR are becoming cost-effective for improving the clinical outcome in patients with BSIs.

## Discussion

BSIs are common situations which are associated with poor outcomes, especially in cases of sepsis/septic shock, immune deficiency, and delayed adequate antimicrobial management. Many septic patients are initially treated in the ED, where rapid administration of targeted antibiotics drastically improve prognosis. Unfortunately, traditional BC are too tardive to support early recognition of sepsis and rapid therapy, which suggests that there is room for improvement. In this study, we assessed the diagnostic performance of ddPCR in detecting suspected sepsis patients in ED clinical practice. Based on the current study, the ddPCR assay exhibited high conformance with the BC method in detecting BSIs. Compared with traditional BC, the ddPCR assay could detect more positive cases and pathogen species for guiding clinical treatment, and greatly shorten the turnover time as well as reduce healthcare costs. Besides, we found the pathogen load of ddPCR was positively correlated with inflammation, coagulation and illness severity associated biomarkers and had the potential to predict a poor clinical outcome. In sum, we firstly determined the efficiency and effectiveness of ddPCR in the quick identification of pathogen and initiation of pathogen-oriented therapy in the ED, which might aid the improvement of management of sepsis and septic shock in the ED.

Until now, BC remains the gold standard assay for the detection of pathogen in the blood stream in the ED. However, the positive ratio of BC is quite low, and it usually takes 24–72 hours or more to isolate disease-causing pathogens (Klouche and Schröder, 2008). Recently, as an emerging flexible and universal platform with high sensitivity and excellent accuracy, ddPCR has been reported to be increasingly used in multiple clinical situations including BSIs but this was at small-scale and not routinely used in the urgent ED environment (Caswell et al., 2020; Hu et al., 2021; Sorbini et al., 2021; Boldrin et al., 2022; Wu et al., 2022). Our study focused on patients presenting at ED with suspected of BSI screened by MSS score more than 2 which had been verified to predict positive BC in the ED in previous studies (Nestor et al., 2021). We designed the multiplex ddPCR panel according to the global and local pathogen epidemiological data which covers the common pathogens of bacteria, virus, and fungi in the ED. Our findings were basically in accordance with the previous studies which suggested ddPCR exhibited superior performance in detecting various infections as well as offered faster turnaround time about 3–4 hours compared to BC (Hu et al., 2021; Shao et al., 2022; Wu et al., 2022; Lin et al., 2023). A recent prospective study enrolled 438 plasma samples from 150 ICU patients, documented the total diagnostic sensitivity rose from 9.1% (40/438) by BC alone to 41.1% (180/438) by ddPCR (Wu et al., 2022). Afterwards, in a prospective, observational, single-center investigation with 122 plasma samples from 169 suspected BSIs patients from the department of infectious diseases, the ED, and the ICU, Lin et al. observed that BC and ddPCR positive rate was 11.27% and 30.28%, respectively (Lin et al., 2023). In contrast to those two studies, the superiority in our cohort is significant as the ddPCR positive rate was 48.13%, which was much higher than the rate of 6% for BC. Among the 18 BC+ cases, 88.8% of which were identified by ddPCR with only 2 missed. The positive rate of BC in our study was relatively lower than previous reports, which may be due to the complexity of emergency patients, and the severity of the disease was not so severe as that in ICU. In consideration of the low positive rate of BC in our study, we define positive ddPCR as identifying same pathogen with BC, another microorganism test, the patient’s condition improved after the treatment of ddPCR pathogen or infection indicators correspond to ddPCR. Therefore, the ddPCR testing showed a potential advantage over BC in terms of an overall sensitivity of 88.89% and specificity of 55.61%. We further found that the optimal diagnostic power for quantifying BSI through ddPCR is achieved with a copy cutoff of 155.5 according to Youden index at maximum. On the other hand, in our research, ddPCR exhibited a relatively high sensitivity in suspected sepsis patients in spite of the extremely low levels of microorganisms in the blood or past antibiotic therapy which may aid in prompt identification of the potential candidates. It is worth mentioning our novel finding that we discovered that in patients with liver abscess, ddPCR detection efficiency was highly sensitive and specific. The clinical symptoms and signs of patients with liver abscess are often atypical, which can cause misdiagnosis or missed diagnosis in clinical environment especially in ED (Kaplan et al., 2004). The long cycle of BC and pus culture is not conducive to the early diagnosis of pathogenic bacteria. Our data suggested that ddPCR might have the ability to detect some recessive BSIs of diseases with a low positive rate of BC as liver abscess which need further large-scale research.

It is worth noting that nearly 10–40% of negative BCs were found to be positive using multiplex molecular approaches during previous studies (Wallet et al., 2010; Peker et al., 2018). Consequently, we concentrated on the interpretation of discordant ddPCR+ results and considered whether BC-/ddPCR+ result represents a real BSI under circumstance of additional factors, including epidemiological, clinical, and laboratory results. Importantly, from the perspective of detailed clinical circumstances, the majority of the discordant results were either probable or possible BSIs. The possible reasons might be accounted for the presence of nonviable, nonproliferating, or transient or intermittent bacteremia, intracellular organisms within circulating phagocytic cells, inhibition of bacterial growth by antibiotics, or possible contamination (Wu et al., 2022). Moreover, organisms identified by microbial cfDNA which are judged as possible or unlikely causes of the sepsis alert included reactivated herpesviruses, chronic infections, microorganisms likely to be commensal organisms or subclinical colonization and possible causes of non-sepsis-related acute infection. Taken together, the sensitivity and specificity of ddPCR were higher than BCs, therefore, it’s indispensable to take ddPCR as an add-on complementary assay to the conventional BC method for identifying the possible causative pathogens and related AMR genes for BC-negative septic patients in emergency department practices.

Another strength of applying ddPCR is to detect multiple bacteria and AMR genes, which could guide the anti-inflammatory treatment. Polymicrobial BSI, defined as the presence of at least two distinct bacteria detected from the BCs or ddPCR, has been recorded more frequently, with rates ranging from 5% to 38% of all BSI events (Hochstein et al., 1965; Kiani et al., 1979; Reuben et al., 1989). Hospitalized patients with polymicrobial BSIs had mortality that varied from 21% to 63%, which is almost twice as high as those of patients with monomicrobial infections (Zhang et al., 2019). The ddPCR assay is more likely to detect polymicrobial BSIs because it eliminates the potential bias produced by preferred amplification and allows detection of pathogens at low quantities when compared to BC. Polymicrobial BSIs were discovered in 7% (15/214) of the patients diagnosed by ddPCR in our current investigation, which was similar to earlier findings (**Supplementary Table S9**) (Lin et al., 2010). Furthermore, our findings illustrated the potential benefit of using ddPCR assays to direct antimicrobial therapy while keeping track of polymicrobial BSIs. The growing number of bacteria with AMR has made clinical anti-infective treatment more difficult. Traditional AST limits timely antibiotics administration and infection control. The current ddPCR in our study, with blaKPC, mecA, blaOXA-48, blaNDM, blaMP, vanA, vanM, seven frequent target AMR genes in one test panel, could directly identify these genes from whole blood in around 3 hours. Notably, prompt and concomitant detection of AMR genes and microbial/polymicrobial infection sources by ddPCR might optimize patient outcomes and allow for better monitoring of resistance mutations (Abram et al., 2020; Hu et al., 2021). In our study, 5 blaKPC, 2 blaNDM, and 10 mecA genes were detected, and blaKPC might be carried by Klebsiella pneumoniae, blaNDM by Klebsiella pneumoniae as well as mecA by Staphylococcus aureus. While no proper causative pathogens were identified for some of blaNDM and mecA genes in several samples. Because the AMR gene identified by ddPCR assay is not from isolated pathogens, which is different from traditional culture-based methods, further research is needed to investigate the causes of mismatches between pathogens and AMR genes. Overall, the data from our investigation and the other clinical trials indicate that the ddPCR test, either alone or in combination with other techniques, can offer a strong platform for early antimicrobial medication start and quick detection of BSIs. However, additional detection panels are required to prevent false-negative findings brought on by the relatively small detection range of ddPCR.

Owing to the clear predominance of bacterial and fungal infections in the context of patients with BSIs, screening for viral infections is rarely part of routine diagnostics. Among all the identified virus by ddPCR, EBV had a substantially higher positive rate than others, which was an interesting finding. EBV, as the first human tumor virus expressing virus cancer genes and immortalizing infected lymphocytes, resides in humans to establish a long-term latent infection and is associated with a variety of human diseases including hematologic malignancies. The levels of viraemia might be considered to be a useful biomarker of immunosuppression, guiding immunotherapy and monitoring disease progression and response to therapy. Our findings were in line with a newly report documented the performance of ddPCR in the children with suspected BSI, with a total of nine viruses including seven EBV out of 44 total identified pathogens by ddPCR (Liu et al., 2023). Our further research discovered that EBV has a link to immunosuppression. 35 (50.72%) were found to have concurrent COVID-19 infection and 21(30.43%) were accompanied with immunosuppression. in our study. In very sick COVID-19 patients, EBV was quite common and was a cause of mortality (Naendrup et al., 2022). Previous research had indicated that viral DNAemia was widespread in severe sepsis, and EBV was the most frequently reactivated virus in plasma in septic patients (Walton et al., 2014; Ong et al., 2017; Mallet et al., 2019). Mallet recently demonstrated that viral DNAemia was highly correlated with the likelihood of secondary infection, a decline in innate immune function over time, and pre-existing immunosuppression (Mallet et al., 2021). Viral DNAemia is expected to be a possible marker of immunosuppression and infection risk in bigger multi-center trials. Herein, ddPCR might be serving as an early warning indicator of the possibility of viraemia in adults with pre-existing or new onset immunosuppressive disorders.

In addition, to our knowledge, we are the first to report that the copies of pathogens in ddPCR were positively correlated with indicators of inflammation severity as well as poor prognosis. One possible explanation is that more serious inflammation in BSIs often exhibits larger loads of causative agent, contributing to a positive ddPCR results. An increasing copy of microorganism facilitates series of inflammatory response, severity and ultimate prognosis. As shown in our study, we found positive correlations between ddPCR pathogen loads and indicators of inflammation (IL-6, CRP, PCT and NLCR levels), severity (Lac) and prognosis (SOFA, APACHII and 28-day mortality). Importantly, we found that the 28-day mortality rate was 2.215 times higher for those with copies greater than 1263. Therefore, the high pathogen load might indicate the development and severity of sepsis. Sepsis-associated dysfunction of coagulation is quite common which usually manifested as abnormal of traditional markers (PLT, PT, APTT and D-dimer) and four new coagulate indicators (TM, TAT, PIC, and t-PAIC). We further found there existed a positive relationship between the pathogen load and these coagulate markers, which suggested the high pathogen load might initiate the disorder of coagulation and thereby aggravate the clinical situations. As for the expectation of prognosis, indeed, we found that clinical variables alone, including the qSOFA score, were unable to accurately estimate outcomes of patients with critical infection at the time of initial evaluation in the ED. Hence, the ddPCR provides us with a complementary tool.

Most recently, a very few clinical trials had suggested that the ddPCR may facilitate precision antimicrobial stewardship by dynamic monitoring pathogens (Shao et al., 2022; Lin et al., 2023). In line with these reports, the enrolled patients’ condition steadily improved following the clinical escalation of antibiotics, and the load of pathogens and drug resistance genes significantly decreased, according to drug resistance gene detection at the same time. In this work, we discovered that variations in pathogen DNA load and number of species detected by ddPCR had the potential to guide antibiotic therapy in critically sick patients with BSIs. With high sensitivity and specificity, the ddPCR detects more pathogens by reducing reporting time, which can drive the optimization of antibiotic management and the rational de-escalation therapy. As a result, ddPCR facilitates precision antimicrobial stewardship and reduces hospitalization costs ultimately. Besides, in view of new diagnostic technology, we not only objectively evaluate the efficiency of its clinical application, but further analyze its value from the perspective of health economics, namely, determine the economic value of the new method. From our data, we found ddPCR assay, with relative low hospitalization/antibiotic cost, high efficiency as well as high specificity and sensitivity, exhibited higher economic efficiency. As a result, the ddPCR assay can help to save medical resources and make it possible to be widely used in clinical practice. Ultimately, patients with suspected sepsis can benefit from the initial first-hand handling in ED in the future.

This study should be considered in light of its limitations. Firstly, this was single-center prospective research, and our findings require further investigation in a multi-center study with a bigger sample size. In addition, the ddPCR system only covered 18 common isolated pathogens and 5 antimicrobial resistance genes, the results of our investigation should be viewed with caution. This revealed the limitation of ddPCR in pathogen detection due to the panel design. Moreover, detections of viruses and AMR genes were not simultaneously compared with other molecular tests, so the results of virus and AMR genes in this study need further investigation. Finally, we only compared the results of ddPCR with conventional BC results, so multiple detection methods should be added to make the results more convincing.

## Conclusion

This single-center study firstly demonstrated that ddPCR had an overall superior detection rate of potential pathogens compared to BC in patients with suspected BSI, which suggest that ddPCR can be used for sepsis pathogen diagnosis models to guide antibiotic treatment. In a timely manner, ddPCR delivered precise and quantitative load data on the causal pathogen, which in part reflected the severity of the infection and poor outcome. Consequently, ddPCR offers the ability to monitor the patient’s condition in real-time and to give medics an early warning of sepsis in time-urgent clinical situations as ED.

## Data availability statement

The original contributions presented in the study are included in the article/**Supplementary Material**, further inquiries can be directed to the corresponding authors.

## Ethics statement

The studies involving humans were approved by Ethics Committee of Shanghai East Hospital. The studies were conducted in accordance with the local legislation and institutional requirements. The participants provided their written informed consent to participate in this study.

## Author contributions

SJ: Writing – original draft, Investigation. DZ: Software, Formal analysis, Data curation, Writing – original draft. CW: Writing – review & editing, Validation, Methodology. XL: Writing – original draft, Supervision, Resources, Investigation. QY: Writing – review & editing, Resources, Investigation, Data curation. XB: Writing – review & editing, Visualization, Resources, Investigation. TD: Writing – original draft, Methodology, Investigation. GL: Writing – original draft, Methodology, Investigation. YG: Writing – original draft, Investigation, Formal analysis. YY: Writing – original draft, Methodology. BS: Writing – review & editing, Investigation. SX: Writing – original draft, Investigation. XZ: Writing – review & editing, Visualization, Validation, Supervision. LF: Writing – review & editing, Supervision, Project administration, Methodology, Funding acquisition. LT: Writing – review & editing, Supervision, Project administration, Funding acquisition.

## Supplementary material

Supplementary Figure 2 The calibration plot of the nomogram.

Supplementary Figure 3 Comparison of the prognosis between negative and positive groups divided by ddPCR assay.

## Glossary

**Table:**

BSI	Bloodstream infection
ED	Emergency department
ddPCR	Droplet digital PCR
MSS	Modified Shapiro Score
BC	Blood culture
CRP	C-reactive protein
PCT	Procalcitonin
SS	Shapiro score
AST	Antibiotic susceptibility testing
qRT-PCR	Real-time quantitative PCR
NGS	Next-generation sequencing
ICU	Intensive care unit
EDTA	Ethylenediaminetetraacetate
AMR	Antimicrobial resistance
APACHE II	Acute Physiological and Chronic Health Assessment II
MEWS	Modified Early Warning Score
SOFA	Sequential Organ Failure Assessment
SD	Standard deviation
IL	Interleukine
AKI	Acute kidney injury
RRT	Renal replacement therapy
G+	Gram-positive
G-	Gram-negative
PPV	Positive predictive value
NPV	Negative predictive value
AUROC	Area under the receiver operating characteristic
EBV	Epstein-Barr virus
CMV	CytoMegaloVirus
VZV	varicella zoster virus
HSV	Herpes simplex virus
VCA	viral capsid antigen
NLCR	Neutrophil Lymphocyte count ratio
WBC	White blood cell
TNF-a	Tumor Necrosis Factor alpha
tPAIC	tissue Plasminogen Activator-inhibitor Complex
TM	thrombomodulin
PT	ProthrombinTime
APTT	Activated Partial Thromboplastin Time
HLA-DR	Human leukocyte antigen DR
TAT	Thrombin-Antithrombin Complex
PIC	plasminogen antifibrinolytic complex
