# Supplementary material for: Clinical evaluation of droplet digital PCR in the early identification of suspected sepsis patients in the emergency department: a prospective observational study
Source: Front Cell Infect Microbiol. 2024 Jun 4;14:1358801. doi: 10.3389/fcimb.2024.1358801 (PMC11183271; doi:10.3389/fcimb.2024.1358801)
Supplement: Supplementary Figure 2 — The calibration plot of the nomogram. [file Image_2.pdf]

Figure S2

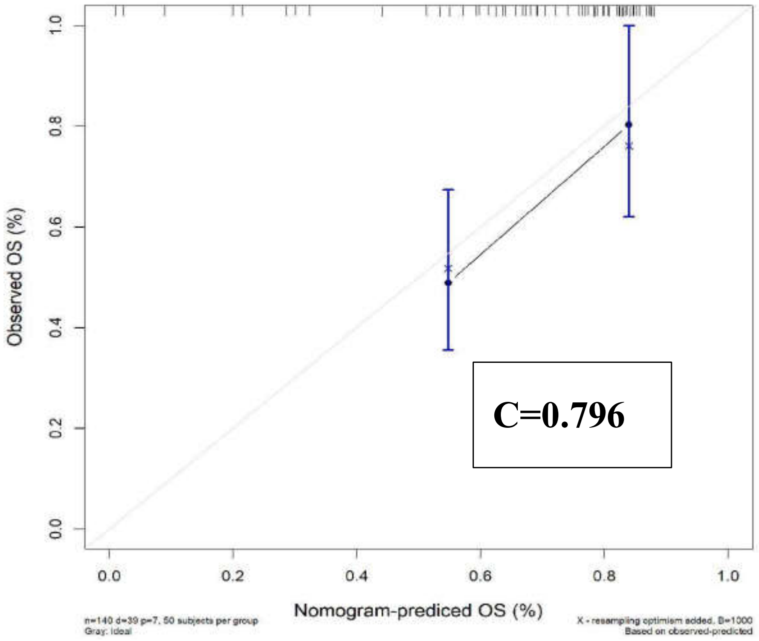

Supplementary Figure 2 The calibration plot of the nomogram. **A** strong statistical consistency in predicting the 28-day mortality caused by BSI, as evidenced by a C value of 0.796. BSI bloodstream infection.
