# Supplementary material for: Clinical evaluation of droplet digital PCR in the early identification of suspected sepsis patients in the emergency department: a prospective observational study
Source: Front Cell Infect Microbiol. 2024 Jun 4;14:1358801. doi: 10.3389/fcimb.2024.1358801 (PMC11183271; doi:10.3389/fcimb.2024.1358801)
Supplement: Supplementary Figure 3 — Comparison of the prognosis between negative and positive groups divided by ddPCR assay. [file Image_3.pdf]

Figure S3

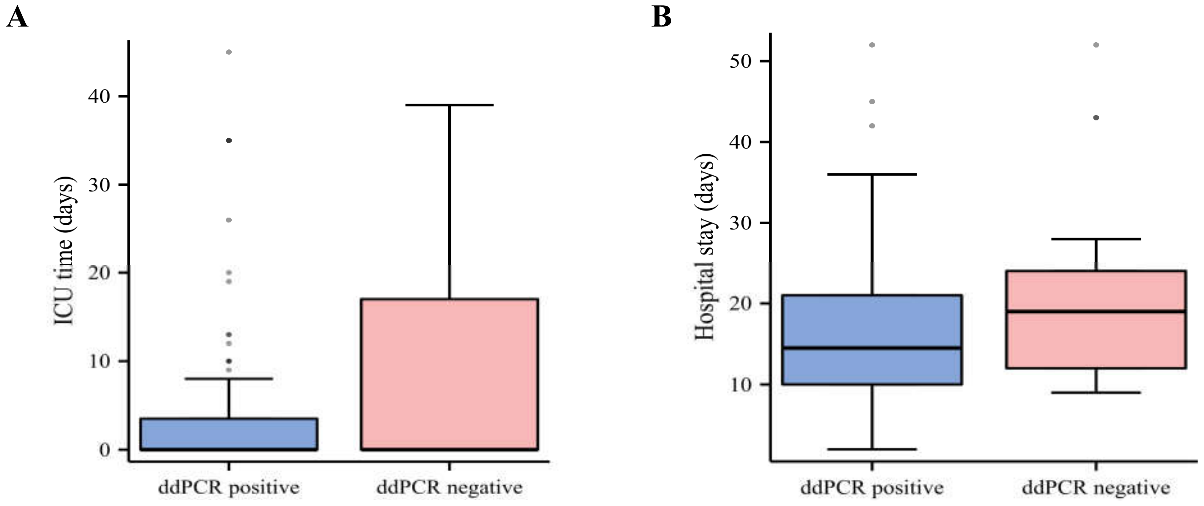

Supplementary Figure 3 Comparison of the prognosis between negative and positive groups divided by ddPCR assay. **A** Comparison of the ICU time between ddPCR positive and ddPCR negative patients. **B** Comparison of the hospital stay between ddPCR positive and ddPCR negative patients.
