# Supplementary material for: Clinical evaluation of droplet digital PCR in the early identification of suspected sepsis patients in the emergency department: a prospective observational study
Source: Front Cell Infect Microbiol. 2024 Jun 4;14:1358801. doi: 10.3389/fcimb.2024.1358801 (PMC11183271; doi:10.3389/fcimb.2024.1358801)
Supplement: Supplementary file 3 [file Table_1.docx]

**Table S1** Panels for detecting pathogens and AMR genes in ddPCR assays

| **Assay panel** | **Target pathogens and AMR genes** |
| --- | --- |
| PilotBac-1 | Pseudomonas aeruginosa,Klebsiella pneumoniae,Escherichia coli,Acinetobacter baumannii |
| PilotBac-2 | Staphylococcus aureus,Enterococcus,Streptococcus,Candida |
| PilotFungi-1 | Pneumocystis jirovecii,Aspergillus,Cryptococcus,Mucor & Rhizopus,talaromyces marneffei |
| PilotVirus-1 | HSV-1/HSV-2, VZV, EBV, CMV |
| PilotAMR-1 | blaKPC, mecA, blaOXA-48,blaNDM,blaIMP,vanA, vanM, |

EBV epstein-barr virus; CMV cytomegalovirus; VZV varicella-zoster virus; HSV Herpes simplex virus

**Table S2** Comparison between BC and ddPCR results within the types of pathogens and AMR

| Case | Blood culture | ddPCR (copy number) | AMR(copy number) |
| --- | --- | --- | --- |
| 1 | Klebsiella pneumoniae | Klebsiella pneumoniae(2408.4) | blaKPC(3726) |
| 6 | Staphylococcus aureus | Staphylococcus aureus(706.5) | / |
| 29 | Bacteroides fragilis | Klebsiella pneumoniae(2480);EBV(535) | blaKPC(921) |
| 33 | Bacteroides fragilis | Klebsiella pneumoniae(1829);streptococcus(165) | blaKPC(1361) |
| 42 | Proteus mirabilis | Klebsiella pneumoniae(184);EBV(74);CMV(85) | blaKPC(86) |
| 55 | Clostridium fusiforme | / | / |
| 117 | Escherichia coli | Escherichia coli(1328)EBV(419copy/ml)CMV(5584copy/ml) |  |
| 144 | Klebsiella pneumoniae | Klebsiella pneumoniae(95)Aspergillus(632)EBV(800)CMV(1958) | blaKPC(90) |
| 151 | Escherichia coli | / | / |
| 157 | Klebsiella pneumoniae | Klebsiella pneumoniae(2821)EBV(232) |  |
| 159 | Streptococcus gordonii | Streptococcus(125) | / |
| 172 | Enterococcus faecium (group D) | Enterococcus(158)EBV(86) | / |
| 190 | Escherichia coli | Escherichia coli(359)EBV(100) | / |
| 206 | Acinetobacter baumannii | Acinetobacter baumannii(4182)Enterococcus(387)Streptococcus(96)EBV(1659) |  |
| 219 | Candida albicans | Candida(428)EBV(184)CMV(68) | / |
| 237 | Escherichia coli | Escherichia coli(448) |  |
| 241 | Enterococcus faecalis | Acinetobacter Baumannii(193)Enterococcus(420)EBV(265) | / |
| 242 | Enterococcus faecalis | Pseudomonas aeruginosa(1252890)Klebsiella pneumoniae(116208)Enterococcus(131625) | / |

EBV epstein-barr virus; CMV cytomegalovirus

**Table S3** The efficacy of ddPCR in diagnosising of Bloodstream infection

| Sample (n= 214) | Sensitivity(%) | Specificity(%) | 95%CI | Cut off value | AUC |
| --- | --- | --- | --- | --- | --- |
| ddPCR+ | 74.2 | 95.7 | (0.753-0.957) | 155.5 | 0.855 |

CI confidence interval; AUC area under curve

**Table S4** Adjustment of antibiotic regimen based on ddPCR assay in 5 [representative](javascript:;) survivors

| Sample | Age(years)/sex | Comorbidities | Initial antibiotics regimen | Blood Culture | First ddPCR assay(copy numbers) | Adjustment of antibiotic regimen based on first ddPCR assay | Second ddPCR assay(copy numbers) | Days to de-escalation therapy | Antibiotic de-escalation regimen |
| --- | --- | --- | --- | --- | --- | --- | --- | --- | --- |
| 29 | 86/male | Gallstone | Biapenem + phosphonomycin + Colistin | Bacteroides fragilis | Klebsiella pneumoniae(2480) blaKPC(921) | Imipenem + Tigecycline + Colistin | Klebsiella pneumoniae(184) blaKPC(86) | 20 | biapenem + phosphonomycin |
| 37 | 71/male | Diabetes mellitus | Imipenem | Negative | Klebsiella pneumoniae(3754) blaIMP(66copy/ml) | Imipenem + Colistin + vancomycin | Klebsiella pneumoniae (57) | 7 | Imipenem |
| 75 | 51/male | Hypertension, Diabetes mellitus, Kidney stone | Phosphonomycin +Tigecycline | Negative | Klebsiella pneumoniae (818) Staphylococcus aureus (51l) blaKPC(193l) mecA(71) | vancomycin + Colistin +Tigecycline | Negative | 14 | phosphonomycin+levofloxacin |
| 141 | 63/male | Hypertension, Diabetes mellitus | Imipenem  +Nemonoxacin | Negative | Staphylococcus aureus(1960) mecA(571) | Imipenem + Nemonoxacin +linezolid | Negative | 13 | Ceftizoxime sodium |
| 144 | 84/female | Valvulopathy, Coronary heart disease, Diabetes mellitus | Imipenem  + Vancomycin + Tigacycline | Negative | Klebsiella pneumoniae(95) Eurotium(632) blaKPC(90) | Colistin  + Fluconazole  + Tigacycline | Negative | 7 | Fosfomycin +Colistin |

**Table S5** List of reasons patients were considered to be immunosuppressed

| **Causes of Immunosuppression** |
| --- |
| Chemotherapy within the last 6 months |
| Hemato-oncological disease |
| High-dose systemic steroid therapy |
| Long-term hemodialysis |
| Immunosuppressive agents and biological agents use |
| neutropenia(absolute neutrophil count＜500/dl) |

**Table S6** Detailed information of liver abscess cases

| Case | Age (years)/ sex | ddPCR assay (copy number)Day1 | Antibiotic regimen | ddPCR assay (copy number) Day3 | abscess cultures （5-7days） | Blood culture(5-7 Day) | Days to deescalation therapy | Antibiotic de-escalation regimen |
| --- | --- | --- | --- | --- | --- | --- | --- | --- |
| 9 | 54 / Male | Escherichia coli(117); Enterococcus（4050） | Meropenem+vancomycin | Negative | Escherichia coli; Enterococcus | Negative | 23 days | Meropenem |
| 62 | 75 / Male | Klebsiella pneumoniae(500) | Imipenem | Negative | Klebsiella pneumoniae | Negative | 7 days | Latamoxef |
| 94 | 74 / Male | Klebsiella pneumoniae(10093) | Meropenem+ Ornidazole | Klebsiella pneumoniae(9881) | Klebsiella pneumoniae | Negative | 7 days | Meropenem |
| 101 | 94 / Male | Escherichia coli(1035) | Imipenem | Negative | Escherichia coli | Negative | 14 days | Piperacillin/tazobactam |
| 151 | 79/ Male | Klebsiella pneumoniae(3976) | Imipenem | Klebsiella pneumoniae(1793) | Klebsiella pneumoniae | Negative | 14 days | Cefperazone-Sulbactam |
| 160 | 52 / Male | Klebsiella pneumoniae(2821) | Imipenem | Klebsiella pneumoniae(594) | Klebsiella pneumoniae | Negative | 10 days | Cefperazone-Sulbactam |
| 254 | 71/Female | Klebsiella pneumoniae(4833) | Imipenem | Klebsiella pneumoniae(1035) | Klebsiella pneumoniae | Negative | 5 days | Piperacillin/tazobactam |

**Table S7** The efficacy of ddPCR in predicting 28-day survival prognosis

| Sample (n= 214) | Sensitivity(%) | Specificity(%) | 95%CI | Cut off value | AUC |
| --- | --- | --- | --- | --- | --- |
| ddPCR+ | 47.2 | 91.4 | (0.614-0.823) | 1263 | 0.718 |

**Table S8** Univariate analysis for patients with BSIs in the development cohort

| Variables in the Equation | B | SE | Wald | df | Sig. | Exp(B) | 95.0% CI for Exp(B) |  |
| --- | --- | --- | --- | --- | --- | --- | --- | --- |
|  |  |  |  |  |  |  | Lower | Upper |
| LG copies | 1.597 | 0.613 | 6.799 | 1 | 0.009 | 4.94 | 1.487 | 16.415 |
| Coronary heart disease | -0.358 | 1.045 | 0.117 | 1 | 0.732 | 0.699 | 0.09 | 5.42 |
| AKI | 1.778 | 1.199 | 2.2 | 1 | 0.138 | 5.917 | 0.565 | 62.007 |
| Combination antibiotic therapy | -0.107 | 1.153 | 0.009 | 1 | 0.926 | 0.898 | 0.094 | 8.6 |
| Immunosuppression | -1.03 | 0.951 | 1.173 | 1 | 0.279 | 0.357 | 0.055 | 2.302 |
| Use of vasoactive drugs | 2.602 | 1.132 | 5.283 | 1 | 0.022 | 13.487 | 1.467 | 123.996 |
| Renal replacement therapy | -2.345 | 2.074 | 1.279 | 1 | 0.258 | 0.096 | 0.002 | 5.58 |
| Mechanical ventilation | -1.089 | 1.134 | 0.922 | 1 | 0.337 | 0.337 | 0.036 | 3.108 |
| PLT | 0.006 | 0.003 | 5.216 | 1 | 0.022 | 1.006 | 1.001 | 1.011 |
| WBC | 0.241 | 0.182 | 1.75 | 1 | 0.186 | 1.272 | 0.891 | 1.816 |
| Neutrophil cell count | -0.152 | 0.166 | 0.837 | 1 | 0.36 | 0.859 | 0.62 | 1.19 |
| Lymphocyte cell count | 0.134 | 0.642 | 0.043 | 1 | 0.835 | 1.143 | 0.325 | 4.027 |
| RDW | 0.541 | 0.169 | 10.258 | 1 | 0.001 | 1.718 | 1.234 | 2.393 |
| CRP | -0.001 | 0.008 | 0.007 | 1 | 0.934 | 0.999 | 0.983 | 1.016 |
| IL6 | -0.002 | 0.001 | 2.616 | 1 | 0.106 | 0.998 | 0.996 | 1 |
| HBP | 0.002 | 0.004 | 0.338 | 1 | 0.561 | 1.002 | 0.995 | 1.01 |
| IL8 | 0.009 | 0.006 | 2.202 | 1 | 0.138 | 1.009 | 0.997 | 1.021 |
| CD8 | -0.001 | 0.003 | 0.208 | 1 | 0.648 | 0.999 | 0.993 | 1.004 |
| C1q | 0.035 | 0.011 | 10.438 | 1 | 0.001 | 1.036 | 1.014 | 1.058 |
| IgM | -3.069 | 1.629 | 3.551 | 1 | 0.06 | 0.046 | 0.002 | 1.131 |
| tPAIC | 0.035 | 0.051 | 0.474 | 1 | 0.491 | 1.035 | 0.938 | 1.143 |
| TM | -0.011 | 0.056 | 0.04 | 1 | 0.842 | 0.989 | 0.887 | 1.103 |
| D-dimer | 0.082 | 0.041 | 3.912 | 1 | 0.048 | 1.085 | 1.001 | 1.176 |
| Fibrinogen | 0.27 | 0.276 | 0.959 | 1 | 0.327 | 1.31 | 0.763 | 2.248 |
| APTT | -0.029 | 0.04 | 0.537 | 1 | 0.463 | 0.971 | 0.899 | 1.05 |
| PT | -0.102 | 0.11 | 0.858 | 1 | 0.354 | 0.903 | 0.728 | 1.121 |
| AGE | -0.033 | 0.041 | 0.634 | 1 | 0.426 | 0.968 | 0.893 | 1.049 |
| MSS score | -0.233 | 0.308 | 0.572 | 1 | 0.449 | 0.792 | 0.433 | 1.449 |
| APACHEII score | 0.124 | 0.104 | 1.442 | 1 | 0.23 | 1.133 | 0.924 | 1.388 |
| SOFA score | 0.026 | 0.231 | 0.013 | 1 | 0.911 | 1.026 | 0.652 | 1.615 |
| MEWS score | 0.981 | 0.292 | 11.256 | 1 | 0.001 | 2.666 | 1.503 | 4.727 |

**Table S9** Detailed information of Polymicrobial detections

| Case | ddPCR assay (copy number) | Blood culture | Other microbial results | Suspected infection site | Consistency with clinical manifestations | Consistency with laboratory results | |
| --- | --- | --- | --- | --- | --- | --- | --- |
| 8 | Escherichia coli(117); Enterococcus（4050） | Negative | Negative | Lower respiratory tract;Urinary tract;Intra-abdominal infection;Abscess | YES | YES |  |
| 17 | Klebsiella pneumoniae(308);Streptococcus(130) | Negative | gram negative bacilli | Lower respiratory tract;Urinary tract | YES | YES |  |
| 25 | Enterococcus(294);Streptococcus(108) | Negative | Enterococcus | Skin and soft tissue | YES | YES |  |
| 33 | Klebsiella pneumoniae(1829);Streptococcus(165);;blaKPC(1361) | Bacteroides fragilis | Negative | Lower respiratory tract;Intra-abdominal infection | YES | YES |  |
| 75 | Klebsiella pneumonia(818);Staphylococcus aureus(51);blaKPC(193)mecA(71) | Negative | Klebsiella pneumonia,Staphylococcus aureus | Urinary tract;Skin and soft tissue | YES | YES |  |
| 91 | Klebsiella pneumonia（9881);Enterococcus（242);Streptococcus（273) | Negative | Klebsiella pneumonia | Intra-abdominal infection | YES | YES |  |
| 101 | Pseudomonas aeruginosa（82);Acinetobacter baumannii（124）;Streptococcus（71） | Negative | Negative | Lower respiratory tract;Urinary tract | YES | YES |  |
| 104 | Enterococcus（227）;Candida（9334) | Negative | Candida | Lower respiratory tract | YES | YES |  |
| 144 | Klebsiella pneumonia(95);Aspergillus(632);blaKPC(90) | Klebsiella pneumonia | Klebsiella pneumonia | Lower respiratory tract;Urinary tract | YES | YES |  |
| 148 | Klebsiella pneumonia(3976);Streptococcus(239) | Negative | Negative | Urinary tract;Skin and soft tissue | YES | YES |  |
| 170 | Klebsiella pneumonia(86);Enterococcus(148) | Negative | Klebsiella pneumonia | Urinary tract;Skin and soft tissue | YES | YES |  |
| 185 | Escherichia coli(968);Candida(293) | Negative | Candida | Urinary tract | YES | YES |  |
| 206 | Acinetobacter baumannii(4182);Enterococcus(387);Streptococcus(96) | Acinetobacter baumannii | Acinetobacter baumannii | Lower respiratory tract;Intra-abdominal infection | YES | YES |  |
| 241 | Acinetobacter baumannii（193）;Enterococcus（420） | Enterococcus | Negative | abscess | YES | YES |  |
| 242 | Pseudomonas aeruginosa（1252890）;Klebsiella pneumonia（116208）;Enterococcus（131625） | Enterococcus | Negative | Lower respiratory tract | YES | YES |  |

**Table S10** Result of microorganisms that are positive for ddPCR but negative for clinical diagnosis

| number | ddPCR assay (copy number) |
| --- | --- |
| 18 | Acinetobacter baumannii(59) |
| 83 | Enterococcus(70);EBV(111);CMV(90) |
| 93 | Streptococcus(62) |
| 138 | Streptococcus(81) |
| 202 | meA(60) |
| 220 | Enterococcus(70） |
| 229 | Acinetobacter baumannii(103) |
| 234 | Acinetobacter baumannii(60)Cryptococcus(63) |
| 159 | Streptococcus(125) |
| 211 | EBV(100) |
