# Supplementary material for: Clinical evaluation of droplet digital PCR in the early identification of suspected sepsis patients in the emergency department: a prospective observational study
Source: Front Cell Infect Microbiol. 2024 Jun 4;14:1358801. doi: 10.3389/fcimb.2024.1358801 (PMC11183271; doi:10.3389/fcimb.2024.1358801)

**Representative cases of liver abscess (Supplementary Fig.1)**

First of all, history and physical signs are important in the identification of liver abscess. Such as sudden high fever, liver pain, chills and local liver tenderness and percussion pain. Patients with these symptoms are suspected of liver abscess, followed by obtaining two sets of blood cultures as well as at least 2 mL whole blood samples (EDTA blood collection tubes) synchronously for BSI diagnosis. Subsequently, laboratory tests such as significantly increased white blood cell count, CRP, SAA as well as elevated aminotransferase and so on could be a manifestation of liver abscess. Finally, auxiliary inspection including B ultrasound and CT are also important for diagnosis. Imaging was characterized by the intrahepatic cystic lesion with clear boundaries and surrounded with inflammatory edema as well as pleural reaction. Bacteria were isolated from the fluid of liver abscess and drug sensitivity test was performed to identify the pathogen after liver abscess liquefies and puncture.

The detailed clinical characteristics presented in No.3 of Table S6. A 74-year-old male patient presented to the emergency department with symptoms of fever and persistent pain in the hepatic region. Following laboratory tests and assessment of vital signs, the possibility of liver abscess was considered, with MSS score≥2. To expedite the identification of pathogens, BC and ddPCR techniques were performed simultaneously. Klebsiella pneumoniae carbapenemases (KPC)-producing Klebsiella pneumoniae (10093copies/ml) was detected by ddPCR within 4 h of the arrival at the emergency department. Subsequently the patient received treatment consisting of meropenem 0.5 g q8h coupled with ornidazole 50 mg qd. On the 3rd day of admission to the emergency department, the quantity of K. pneumoniae(9881copies/ml) detected by the second ddPCR assay was lower compared to previous measurements. However, the patient was unable to undergo abscess puncture due to an immature abscess as observed by ultrasonography. On the 7th day, the third ddPCR result indicated that load of Klebsiella pneumoniae(519 copies/ml) decreased further with the implementation of antibiotic de-escalation therapy(meropenem 0.5 g q8h only). Meanwhile, the blood culture came out with negative results. On the 10th day, the patient's liver abscess matured and drained with puncture, which was subsequently sent for culture. The bacterial culture of pyogenic fluids came out on day 14 and confirmed the presence of K. pneumoniae. At the same time, the patient’s symptoms improved, and his body temperature recovered normal. All antibiotics were discontinued, and the patient was released from the medical facility on the 16th day. Looking back on the treatment process of this case. The pus culture necessitates waiting for the abscess maturation, resulting in delayed etiological findings. Failure to promptly administer targeted antibiotics may result in the deterioration of diseases rapidly, ultimately leading to septic shock. Therefore, this case highlights the significance of employing highly sensitive diagnostic techniques, such as ddPCR, for the analysis of liver abscesses. The ddPCR enables more rapid (4 hours vs. 7 days) and precise identification of pathogens compared to traditional BC. Besides, ddPCR can provide early initiation of accurate antibiotic therapy and guide antibiotic de-escalation therapy.

**Supplementary Figure 1** The representative quantitative value of ddPCR in the dynamic surveillance of liver abscess from one patient


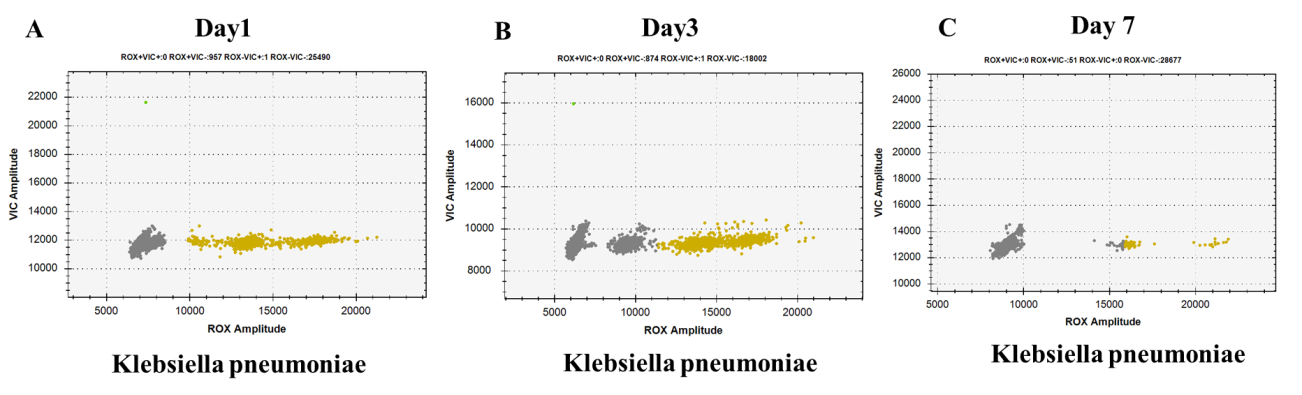


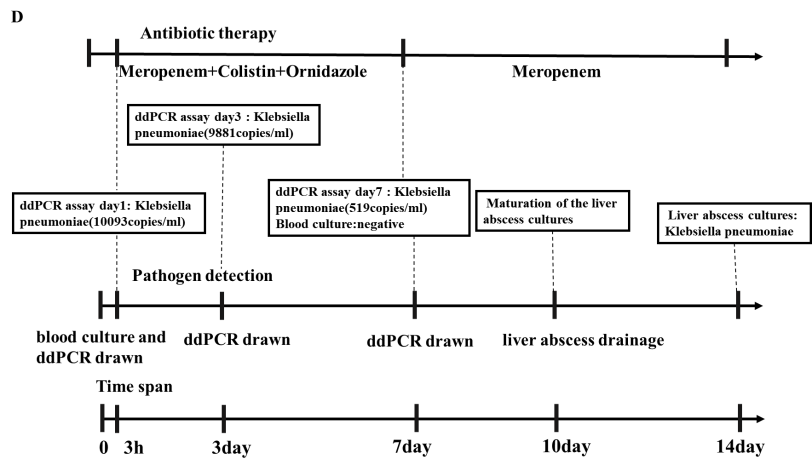

Supplement: Supplementary file 4 [file DataSheet_1.docx]
